# Disruption of Lipid Raft Function Increases Expression and Secretion of Monocyte Chemoattractant Protein-1 in 3T3-L1 Adipocytes

**DOI:** 10.1371/journal.pone.0169005

**Published:** 2016-12-28

**Authors:** Juu-Chin Lu, Yu-Ting Chiang, Yu-Chun Lin, Yu-Tzu Chang, Chia-Yun Lu, Tzu-Yu Chen, Chia-Shan Yeh

**Affiliations:** 1 Department of Physiology and Pharmacology, Chang Gung University, Taoyuan, Taiwan; 2 Graduate Institute of Biomedical Sciences, Chang Gung University, Taoyuan, Taiwan; 3 Division of Endocrinology and Metabolism, Department of Internal Medicine, Chang Gung Memorial Hospital, Linkou, Taiwan; 4 Department of Biomedical Sciences, College of Medicine, Chang Gung University, Taoyuan, Taiwan; Tohoku University, JAPAN

## Abstract

The adipocyte is unique in its capacity to store lipids. In addition to triglycerides, the adipocyte stores a significant amount of cholesterol. Moreover, obese adipocytes are characterized by a redistribution of cholesterol with depleted cholesterol in the plasma membrane, suggesting that cholesterol perturbation may play a role in adipocyte dysfunction. We used methyl-β-cyclodextrin (MβCD), a molecule with high affinity for cholesterol, to rapidly deplete cholesterol level in differentiated 3T3-L1 adipocytes. We tested whether this perturbation altered adipocyte secretion of monocyte chemoattractant protein-1 (MCP-1), a chemokine that is elevated in obesity and is linked to obesity-associated chronic diseases. Depletion of cholesterol by MβCD increased MCP-1 secretion as well as the mRNA and protein levels, suggesting perturbation at biosynthesis and secretion. Pharmacological inhibition revealed that NF-κB, but not MEK, p38 and JNK, was involved in MβCD-stimulated MCP-1 biosynthesis and secretion in adipocytes. Finally, another cholesterol-binding drug, filipin, also induced MCP-1 secretion without altering membrane cholesterol level. Interestingly, both MβCD and filipin disturbed the integrity of lipid rafts, the membrane microdomains enriched in cholesterol. Thus, the depletion of membrane cholesterol in obese adipocytes may result in dysfunction of lipid rafts, leading to the elevation of proinflammatory signaling and MCP-1 secretion in adipocytes.

## Introduction

The physiological function of adipose tissue is to store lipids. Excess energy intake results in the storage of triglyceride, a neutral lipid composed of esterified fatty acids and glycerol, in the lipid droplet of adipocytes. When energy expenditure exceeds energy intake, stored triglycerides are hydrolyzed to generate and release fatty acids to supply the energy demand of other tissues. In addition to triglycerides, other lipid species such as cholesterol are also present in the lipid droplet of the adipocytes. In fact, the adipose tissue is the largest pool of cholesterol in the human body [1, 2]. Moreover, the cholesterol found in the adipocytes is mostly in its free form, which distinguishes it from the cholesterol esters stored in the steroid hormone-producing adrenal cortical cells or cholesterol-laden foam cells [3]. Thus, the unique form and significant amount of cholesterol in adipocytes suggest a possible role for cholesterol in regulating adipocyte function. Indeed, both cholesterol and triglycerides are elevated and accumulated in hypertrophied adipocytes [2, 4]. However, cholesterol is redistributed in these obese adipocytes, resulting in the depletion of cholesterol in the plasma membrane of hypertrophied adipocytes [1–3, 5–7]. Therefore, cholesterol imbalance may have an impact on adipocyte function during obesity or other disease conditions. Currently, the role of cholesterol in adipocyte function is not fully understood.

In addition to being a structural component that modulates the fluidity of the plasma membrane, cholesterol has many other cellular functions. Lipid rafts, the specific membrane microdomains that are enriched in cholesterol and sphingolipids, as well as caveolae, a subset of lipid raft domains consisting of the structure protein caveolin, are involved in many cellular processes, such as signal transduction [8], endocytosis [9], and cellular trafficking [10]. In adipocytes, depletion of membrane cholesterol, which disrupts the integrity of lipid rafts and caveolae [11], has been shown to affect the translocation of the insulin-responsive glucose transporter 4 (GLUT4) [12] and insulin signaling [13]. However, the role of cholesterol levels and lipid rafts in other adipocyte functions remains largely unknown.

Adipose tissue is now recognized as an endocrine organ that actively secretes peptide hormones and cytokines, which are collectively called adipokines. Abnormal secretion of adipokines has been linked to the dysfunction of adipose tissue and many chronic diseases. Among the adipokines secreted by adipocytes, monocyte chemoattractant protein-1 (MCP-1) is a chemokine that plays an important role in the recruitment of monocytes and T lymphocytes to the sites of inflammation. MCP-1 is expressed and secreted by adipocytes [14] and other cell types such as macrophages, smooth muscle cells, and endothelial cells [15]. The expression and secretion of MCP-1 are elevated in obesity [14, 16], and these higher levels have been linked to obesity-associated chronic diseases such as insulin resistance, diabetes, or cardiovascular disease (for reviews, [17, 18]). Factors that are elevated in obesity, such as tumor necrosis factor α (TNFα) and interleukin 6 (IL6), increase the expression and secretion of MCP-1. In contrast, factors that prevent insulin resistance such as adiponectin and the anti-diabetic drug thiazolidinediones, down-regulate the secretion of MCP-1 in adipocytes [17]. These observations suggest a link between the MCP-1 level, obesity, and insulin resistance. In this study, we added methyl-β-cyclodextrin (MβCD), a cyclic oligosaccharide with high affinity to cholesterol, in culture medium to rapidly deplete cholesterol in differentiated 3T3-L1 adipocytes [19] and measured the effect on MCP-1 secretion in adipocytes. Interestingly, MβCD treatment increased MCP-1 expression and secretion in 3T3-L1 adipocytes through activation of nuclear factor kappa-light-chain-enhancer of activated B cells (NF-κB). However, the disturbance of lipid raft integrity rather than the decrease of cellular or membrane cholesterol may account for the MβCD-mediated increase of MCP-1 expression and secretion. Thus, the depletion of membrane cholesterol in obese adipocytes may lead to the disturbance of lipid rafts, which results in elevated proinflammatory responses and production of proinflammatory cytokines and chemokines in obesity.

## Materials and Methods

### Chemicals and Reagents

MβCD (#C4555), water-soluble cholesterol (WSCL, #C4951), filipin complex (#F9765), BMS-345541 (#B9935), SP600125 (#S5567), and SB203580 (#S8307) were purchased from Sigma Chemical (St. Louis, MO). Recombinant murine TNFα (No. 410-MT) was from R & D Systems (Minneapolis, MN). Polyclonal antibodies against phospho-ERK1/2 (Thr202/Tyr204, #4377), ERK1/2 (#9102), NF-κB p65 (#8242), phospho-IκBα (Ser32, #2859), IκBα (#9242), MEK1/2 (#9122), caveolin-1 (#3267), and MCP-1 (#2029) were from Cell Signaling Technology (Beverly, MA). Anti-α-tubulin (#T5168) antibodies were from Sigma Chemical (St Louis, MO). Anti-flotillin antibodies (#610820) were purchased from BD Biosciences (San Jose, CA). Anti-IKKβ antibodies (#05–535) were from Millipore (Billerica, MA). GSK1120212 was purchased from Selleck Chemicals (Houston, TX).

### Cell Culture, Differentiation, and Electroporation

3T3-L1 fibroblasts (#CL-173, from American Type Culture Collection, Manassas, VA) were cultured and differentiated as described previously [20]. Briefly, cells were grown in the growth media [Dulbecco’s modified Eagle medium (DMEM) supplemented with 4.5 g/L glucose, 10% fetal bovine serum, 1% glutamine, and 0.5% penicillin/streptomycin]. Differentiation was induced in post-confluent cells with growth media containing 500 μM isobutylmethylxanthine, 0.2 μM dexamethasone, and 2.5 μg/ml insulin for 3–4 days, and cells were replenished with growth media every 3–4 days. Experiments were performed in adipocytes 12–16 days post differentiation.

Electroporation of 3T3-L1 adipocytes was performed with cells on 10–12 days post differentiation. Differentiated 3T3-L1 adipocytes were electroporated at 200 V and 950 μF with 2 nmole siRNA using a Gene Pulser Xcell electroporator (Bio-Rad, Hercules, CA) and plated onto 12- or 24- well plates for experiments. The siRNA sequences were as follows: MEK1 and 2 (MEK1/2), 5’-AGU CGG ACA UCU GGA GCA U-3’, or 5’-CAG UCG GAC AUC UGG AGC A-3’; IKKβ, 5’-CGA CAG GAG CUC AGC CCA A-3’, or 5’-GGA CAU CGU UGU UAG UGA A-3’; caveolin-1, 5’-AAC CAG AAG GGA CAC ACA G-3’ [21].

### RNA Analysis

Total cellular RNA was isolated and purified using TRIzol reagent (Ambion, Austin, TX) according to the manufacturer’s instructions. First strand cDNA was synthesized from 1 μg total RNA using high-capacity cDNA RT kits (Applied Biosystems). SYBR Green PCR was performed using the MiniOpticon real-time PCR detection system (Bio-Rad, Hercules, CA). The following primers were used for PCR: MCP-1 forward 5’-AGG TCC CTG TCA TGC TTC TG-3’, reverse 5’-GCT GCT GGT GAT CCT CTT GT-3’; 36B4 forward 5’-GCG ACC TGG AAG TCC AAC TAC-3’, reverse 5’-ATC TGC TGC ATC TGC TTG G-3’. Gene expression levels were calculated after normalization to the housekeeping gene 36B4 using the ΔΔCT method as described by the manufacturer and expressed as relative mRNA levels compared with the control.

### Enzyme-Linked Immunosorbent Assay (ELISA)

Adipocytes were placed into growth medium or DMEM with 0.1% BSA containing the treatments. Media were collected at indicated time points and centrifuged at 14,000 rpm for 5 min to remove floating cells. Conditioned medium was assayed for mouse MCP-1 using an ELISA kit (Biosource, Camarillo, CA) following the manufacturer's protocol. The cell lysates were collected and protein concentrations were measured and used to normalize MCP-1 secretion.

### Membrane Fractionation

Differentiated 3T3-L1 adipocytes grown on two ten-centimeter dishes were scraped and collected in a 50-ml tube. After centrifugation at 3,500 rpm for 15 min, the pellet was resuspended in 15 ml lysis buffer (10 mM Tris-HCl, pH 7.5, 10 mM NaCl, 1 mM MgCl_2_, 1% aprotinin). After another centrifugation, the pellet was resuspended in lysis buffer, diluted with resuspension buffer (TSNa buffer, 20 mM Tris-HCl, pH 7.5, 50 mM NaCl, 250 mM sucrose, and 1% aprotinin) and was subjected to homogenization. Unbroken cells or nuclei were removed by centrifugation at 3,500 rpm for 15 min, and the supernatant was transferred to a Ti45 rotor tube. After centrifugation at 35,000 rpm (120,000 x *g*) at 4°C for 1 h, the supernatant was stored for cholesterol measurement or was precipitated by trichloroacetic acid for Western analysis. The membrane pellet was resuspended with storage buffer [TSNa buffer containing 10% (v/v) glycerol and 1% aprotinin] using a 23-gauge syringe needle.

### Measurement of Cellular and Cholesterol Levels

Cellular and plasma membrane cholesterol levels were determined using the Amplex Red Cholesterol Assay Kit (Molecular Probes/Life Technologies, Grand Island, NY) according to the manufacturer’s instructions. In brief, 50 μl sample was mixed with 50 μl Amplex Red Reagent containing 2 U/ml HRP, 2 U/ml cholesterol oxidase, and 0.2 U/ml cholesterol esterase and was incubated for 30 min at 37°C. Fluorescence was measured by the TECAN infinite M200 PRO microplate reader (Mannedorf, Switzerland) using excitation and emission at 530 and 590 nm, respectively.

### Western Blot Analysis

Western blot analysis was performed as previously described [20]. Briefly, cells were extracted with RIPA lysis buffer [50 mM HEPES, pH 7.4, 1% NP-40, 150 mM NaCl, 1 mM EDTA, 1 mM phenylmethylsulfonyl fluoride, 1% protease inhibitor cocktail (Sigma #P2714), 1 mM sodium orthovanadate, 1 mM sodium fluoride]. Twenty microliters of cellular protein lysate was electrophoresed through standard Laemmli SDS polyacrylamide gels (8–12% gels), and then transferred to polyvinylidene fluoride membranes. Membranes were blocked for 1 h in 5% BSA or non-fat milk in TBST (100 mM Tris-HCl, pH 7.5, 150 mM NaCl, and 0.1% Tween 20) and then incubated in primary antibodies at 4°C overnight. Membranes were washed three times with TBST and then incubated with secondary antibodies in 5% milk or non-fat milk in TBST at room temperature for 1 h. Membranes were washed three times with TBST, and then signals were visualized by enhanced chemiluminescence using the ChemiDoc XRS^+^ imaging system (Bio-Rad, Hercules, CA).

### Lipid Raft Fractionation

Lipid raft fractionation was performed as described previously [22]. In brief, differentiated 3T3-L1 adipocytes grown from two ten-centimeter dishes were washed and collected. After centrifugation at 100 x *g* for 5 min, the cell pellet was lysed in 1 ml TNE/Triton X-100 buffer (25 mM Tris-HCl, pH 7.4, 150 mM NaCl, 5 mM EDTA, and 1% Triton X-100) on ice for 20 min. Cell lysate was homogenized with eight strokes of a Dounce homogenizer and was transferred to an ultracentrifuge tube. The lysate was brought to 40% sucrose by adding equal volume of 80% sucrose. A 5% to 30% linear sucrose gradient was layered over the lysate. Samples were centrifuged at 200,000 x *g* at 4°C for longer than 15 h in a Beckman SW41 rotor. Each 1 ml fraction from the top to the bottom was transferred and collected.

### Statistics

All of the data are presented as the mean ± S.E. Differences between the means of two groups were evaluated for statistical significance with paired or unpaired Student’s two-tailed *t*-tests. A *p* value cut-off of 0.05 was considered statistically significant (InStat 3, GraphPad).

## Results

### Cholesterol Depletion Increases MCP-1 Secretion in 3T3-L1 Adipocytes

**Fig 1:**
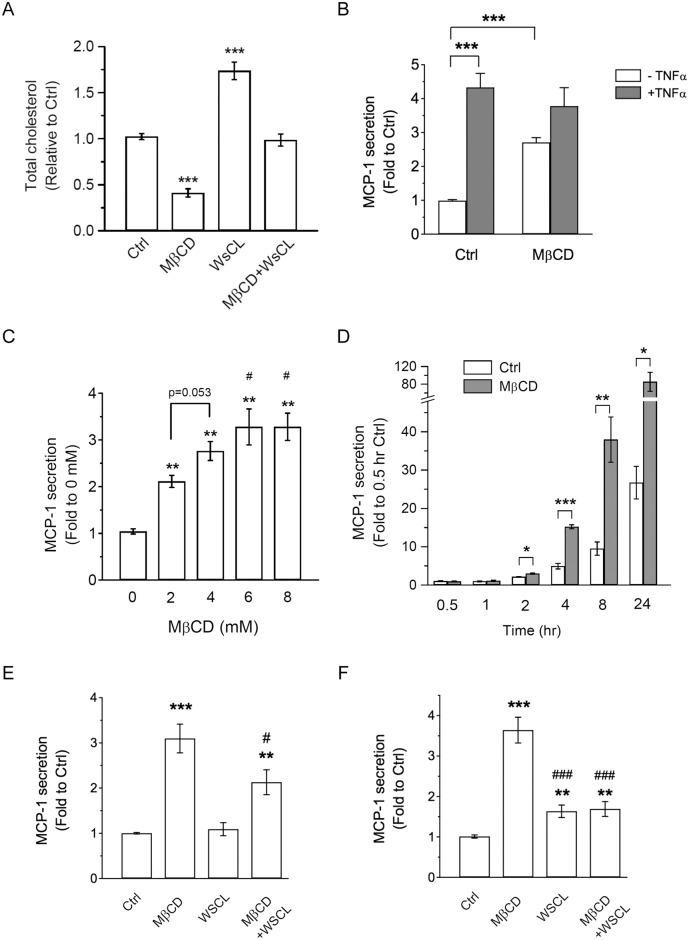
MβCD treatment increases MCP-1 secretion in 3T3-L1 adipocytes. (A) 3T3-L1 adipocytes were untreated (Ctrl) or treated with 4 mM MβCD, 250 μg/ml WSCL, or both for 4 h. Cellular lysate was collected and cholesterol levels were determined. (B) 3T3-L1 adipocytes were untreated (Ctrl) or treated with 4 mM MβCD, together without or with 1 ng/ml TNFα for 4 h. MCP-1 secretion in the media was determined. Each point represents the mean ± S.E. of three independent experiments. Asterisks denote significant differences (***p<0.001). (C) 3T3-L1 adipocytes were treated with increasing MβCD doses (0, 2, 4, 6, 8 mM) for 4 h. MCP-1 secretion was determined. Each point represents the mean ± S.E. of three independent experiments. **p<0.01, compared with 0 mM MβCD treatment. #p<0.05, compared with 2 mM MβCD treatment. (D) 3T3-L1 adipocytes were untreated (Ctrl) or treated with 4 mM MβCD for different time points (0.5, 1, 2, 4, 8, or 24 h). MCP-1 secretion was determined. Each point represents the mean ± S.E. of three independent experiments. Asterisks denote significant differences (*p<0.05, **p<0.01, ***p<0.001). (E) 3T3-L1 adipocytes were untreated (Ctrl) or treated with 4 mM MβCD, 250 μg/ml WSCL, or both for 4 h. MCP-1 secretion in the media was determined. Each point represents the mean ± S.E. of eight independent experiments. **p<0.01, ***p<0.001, compared with Ctrl. #p<0.05, compared with MβCD treatment. (F) 3T3-L1 adipocytes were untreated (Ctrl) or treated with 4 mM MβCD, 250 μg/ml WSCL, or both for 24 h. MCP-1 secretion in the media was determined. Each point represents the mean ± S.E. of six independent experiments. **p<0.01, ***p<0.001, compared with Ctrl. ###p<0.001, compared with MβCD treatment.

It has been reported that *de novo* cholesterol synthesis is limited in adipocytes [3]. Consistently, treatment with inhibitors that suppress cholesterol synthesis (mevastatin or U18666A) [7, 23] did not alter cholesterol levels in differentiated 3T3-L1 adipocytes even up to 24 h incubation (S1 Fig). Therefore, we used MβCD, a molecule with high affinity for cholesterol, to rapidly deplete cholesterol in differentiated 3T3-L1 adipocytes [19]. Treatment with MβCD for 4 h greatly reduced cholesterol level in adipocytes (Fig 1A). We tested whether cholesterol depletion affected adipocyte secretion of MCP-1, a chemokine involved in obesity-associated chronic diseases [17, 18]. 3T3-L1 adipocytes were treated without (Ctrl) or with MβCD for 4 hours, and MCP-1 secretion in the medium was measured by ELISA. Interestingly, depletion of cholesterol by MβCD treatment increased MCP-1 secretion in adipocytes (Fig 1B). In obesity, elevated levels of proinflammatory cytokines, such as TNFα, stimulate MCP-1 secretion [17]. Therefore, we also tested whether MβCD treatment affected TNFα-induced MCP-1 secretion. As expected, TNFα treatment greatly increased MCP-1 secretion, which is consistent with the idea that TNFα is a strong proinflammatory inducer of MCP-1 in obesity. Simultaneous treatment of MβCD and TNFα did not suppress nor enhance the TNFα-induced MCP-1 secretion (Fig 1B). Together, these results suggested that depletion of cholesterol by MβCD treatment increased MCP-1 secretion in adipocytes.

To determine the effective MβCD dose needed to increase MCP-1 secretion in adipocytes, we performed a MβCD dose response experiment. 3T3-L1 adipocytes were treated with different MβCD doses (0, 2, 4, 6, 8 mM) for 4 h and MCP-1 secretion was determined. As shown in Fig 1C, a dose-dependent increase of MCP-1 secretion was observed with 4 h MβCD treatment at 2 mM or greater. To test if MβCD-induced MCP-1 secretion is time-dependent, a time course experiment was performed. As shown in Fig 1D, a significant increase of MCP-1 secretion was observed after 2 h MβCD treatment. We also examined whether MβCD treatment induced cytotoxicity in 3T3-L1 adipocytes, and found that only 24 h treatment with MβCD at 7.5 mM or higher reduced cell viability (S2 Fig).

To confirm that the effects of MβCD treatment were due to cholesterol depletion, we supplied water-soluble cholesterol (WSCL) to restore cholesterol levels and reverse the action of MβCD (Fig 1A, and [11, 24, 25]). As shown in Fig 1E, simultaneous treatment with MβCD and WSCL for 4 h partially reversed the MβCD-induced MCP-1 secretion. Moreover, treatment with WSCL for 24 h almost returned MβCD-induced MCP-1 secretion to the Ctrl levels (Fig 1F).

### Depletion of Membrane Cholesterol Increases MCP-1 Biosynthesis

**Fig 2:**
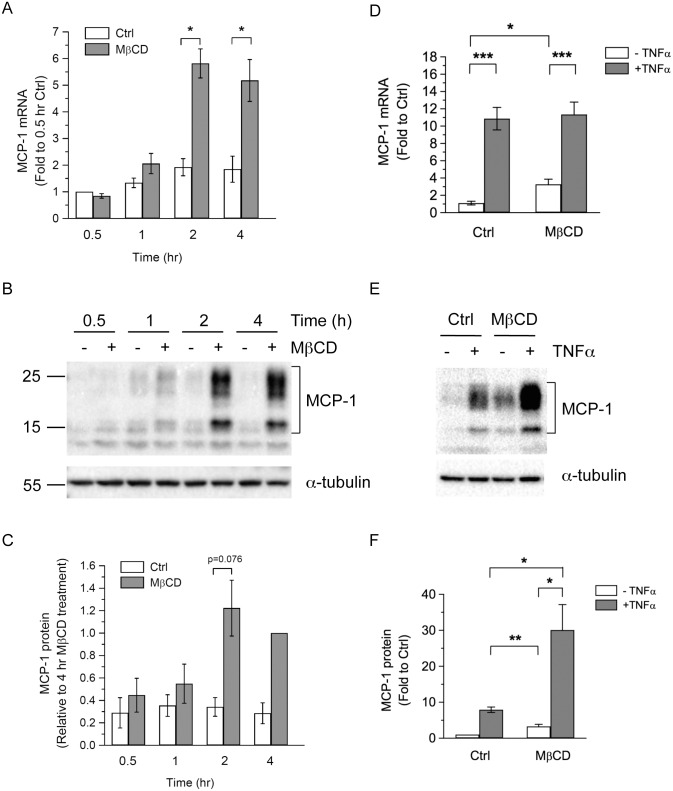
MβCD treatment increases mRNA and protein expression of MCP-1. (A-C) 3T3-L1 adipocytes were untreated (Ctrl) or treated with 4 mM MβCD for 0.5, 1, 2, or 4 h. The levels of mRNA (A) and protein (B,C) were determined by qPCR and Western blot analysis, respectively. Each point represents the mean ± S.E. of at least three independent experiments. Asterisks denote significant differences (*p<0.05). (D-F) 3T3-L1 adipocytes were untreated (Ctrl) or treated with 4 mM MβCD, together without or with 1 ng/ml TNFα for 4 h. The levels of mRNA (D) and protein (E,F) were determined by qPCR and Western blot analysis, respectively. Representative immunoblots from three independent experiments are shown. Each point represents the mean ± S.E. of at least three independent experiments. Asterisks denote significant differences (*p<0.05, **p<0.01, ***p<0.001).

Treatment with the protein synthesis inhibitor cycloheximide reduced the basal MCP-1 secretion and completely blocked MβCD- or TNFα-induced MCP-1 secretion (S3 Fig), suggesting that elevated MCP-1 secretion induced by MβCD treatment may attribute to the increase in MCP-1 biosynthesis. Therefore, we measured the mRNA and protein levels of MCP-1 after MβCD treatment. As shown in Fig 2A, MβCD treatment significantly increased the mRNA levels of MCP-1 in adipocytes starting at 2 h. Similarly, increased MCP-1 protein levels were observed after 2 h MβCD treatment (Fig 2B and 2C).

We also examined the effects of combined treatment with MβCD and TNFα on MCP-1 mRNA and protein levels. While both MβCD and TNFα could induce MCP-1 mRNA levels after 4 h treatment, TNFα treatment induced a much greater increase of MCP-1 mRNA than MβCD treatment (Fig 2D). Simultaneous treatment with MβCD and TNFα did not further increase the MCP-1 mRNA level, compared with TNFα treatment alone (Fig 2D). In contrast, an additive effect was observed in the increase of MCP-1 protein levels when adipocytes were simultaneously treated with MβCD and TNFα (Fig 2E and 2F).

### Depletion of Membrane Cholesterol Increases ERK Phosphorylation

**Fig 3:**
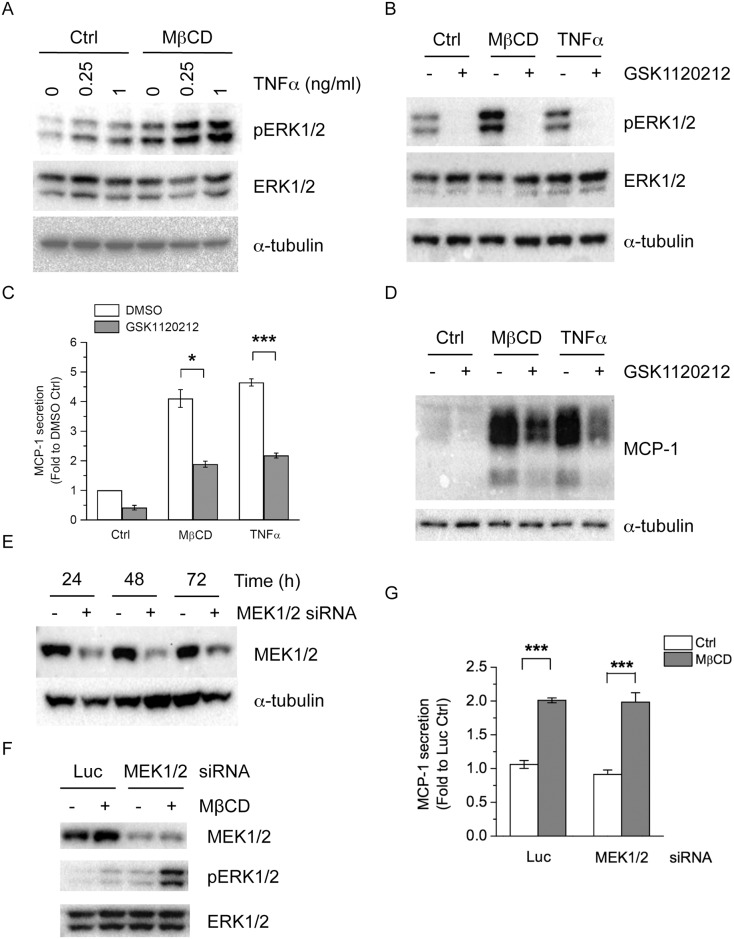
Effects of MEK1/2 inhibition and depletion on MβCD-induced MCP-1 secretion. (A) 3T3-L1 adipocytes were untreated (Ctrl) or pretreated with 4 mM MβCD for 4 h, and then treated with increasing TNFα doses (0, 0.25, 1 ng/ml) for 30 min. (B) 3T3-L1 adipocytes were untreated (Ctrl), or treated with 4 mM MβCD or 1 ng/ml TNFα, together without or with 10 μM GSK1120212 for 4 h. Cellular proteins were solubilized and subjected to SDS-PAGE and Western blot analysis with the indicated antibodies. Representative immunoblots from three independent experiments are shown. (C) 3T3-L1 adipocytes were untreated (Ctrl), or treated with 4 mM MβCD or 1 ng/ml TNFα, together without (DMSO) or with 10 μM GSK1120212 for 4 h. MCP-1 released into the media and protein concentrations of cell lysate were determined. Each point represents the mean ± S.E. of three independent experiments. Asterisks denote significant differences (*p<0.05, ***p<0.001). (D) 3T3-L1 adipocytes were untreated (Ctrl), or treated with 4 mM MβCD or 1 ng/ml TNFα, together without or with 10 μM GSK1120212 for 4 h. Cellular proteins were solubilized and subjected to SDS-PAGE and Western blot analysis with the indicated antibodies. Representative immunoblots from three independent experiments are shown. (E) 3T3-L1 adipocytes were transfected with non-targeting luciferase siRNA (-), or siRNA against MEK1/2 (+). Protein levels of MEK1/2 were determined by Western blotting. (F,G) 3T3-L1 adipocytes were transfected with luciferase (Luc) or MEK1/2 siRNA. 48 h post transfection, cells were untreated (Ctrl) or treated with 4 mM MβCD for 4h. Total and phosphorylation of ERK1/2 were detected (F). MCP-1 released into the media was determined by ELISA (G). Each point represents the mean ± S.E. of three independent experiments. Asterisks denote significant differences (***p<0.001).

The activation of mitogen-activated protein kinases (MAPKs), including extracellular signal-regulated kinase (ERK) [15, 26], c-Jun N-terminal kinase (JNK) [16, 27–29], p38 [15, 29], mediates MCP-1 expression and secretion in many cell systems including adipocytes. To determine the signaling pathways involved in MβCD-induced MCP-1 expression and secretion in adipocytes, we used pharmacological inhibitors directed against each MAPK and tested their effects on MβCD-induced MCP-1 expression and secretion. We also included TNFα treatment for comparison because TNFα is a known inducer of MCP-1 and MAPK signaling pathways. We first determined if MβCD treatment induced phosphorylation of ERK1/2 in 3T3-L1 adipocytes. As shown in Fig 3A, TNFα treatment increased phosphorylation of ERK1/2 in a dose-dependent manner. MβCD treatment also greatly increased ERK1/2 phosphorylation (Fig 3A and 3B). Simultaneously treating cells with MβCD and TNFα led to an additive elevation of ERK1/2 phosphorylation (Fig 3A). Moreover, addition of WSCL, which increased cholesterol levels in adipocytes (Fig 1A), reduced TNFα-induced ERK phosphorylation (S4 Fig). Simultaneously treating cells with MβCD and WSCL reversed MβCD-inducde ERK phosphorylation (S4 Fig). These results demonstrated that MβCD treatment, which depleted cholesterol levels, induced ERK1/2 phosphorylation in adipocytes.

We used GSK1120212, which inhibits mitogen-activated protein kinase kinases (MEKs) that are the upstream kinases to ERK1/2, to test whether ERK1/2 is involved in MβCD-induced MCP-1 secretion. GSK1120212 treatment inhibited basal, MβCD-, or TNFα-induced ERK phosphorylation, consistent with its suppression of MEKs and ERK1/2 (Fig 3B). We then determined if inhibition of MEK1/2 affected MβCD-induced MCP-1 secretion and expression. As shown in Fig 3C and 3D, GSK1120212 treatment not only reduced basal MCP-1 secretion, but also reduced MβCD- and TNFα- mediated increases of MCP-1 secretion (Fig 3C) and expression (Fig 3D).

To confirm the results from pharmacological inhibition, we knocked down MEK1 and MEK2, the cellular targets of GSK1120212 [30] to test if MEK1/2 mediated MβCD-induced MCP-1 secretion. siRNA duplexes were designed in the conserved regions of mouse MEK1 and MEK2 to simultaneously deplete both isoforms in adipocytes. As shown in Fig 3E, MEK1 and MEK2 proteins were greatly depleted after RNA interference. Surprisingly, knockdown of MEK1 and MEK2 did not affect MβCD-induced MCP-1 secretion (Fig 3G). Moreover, chronic depletion of MEK1 and MEK2 increased basal and MβCD-induced ERK phosphorylation (Fig 3F).

To examine if JNK and p38 MAPKs were involved in MβCD-induced MCP-1 secretion and expression in adipocytes, we used SP600125 and SB203580, the inhibitors of JNK and p38, respectively. JNK and p38 MAPK are involved in FFA-induced MCP-1 expression in 3T3-L1 adipocytes [16, 29]. Interestingly, we found that JNK and p38 were involved in TNFα-induced, but not MβCD-induced MCP-1 expression and secretion in 3T3-L1 adipocytes (S5A and S5D Fig).

### Activation of NF-κB Mediates MβCD-induced MCP-1 Secretion

**Fig 4:**
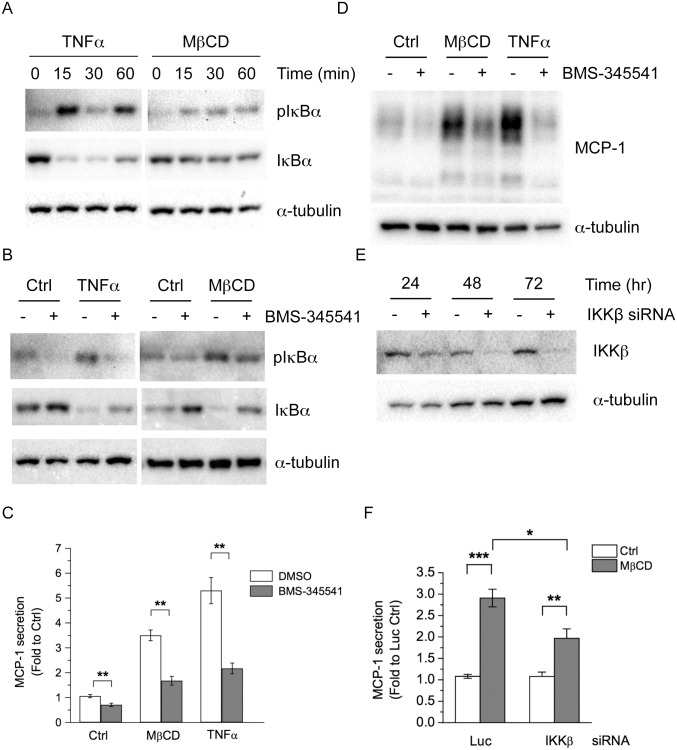
Inhibition of IKK suppresses MβCD-induced MCP-1 expression and secretion. (A) 3T3-L1 adipocytes were treated with 1 ng/ml TNFα or 4 mM MβCD for 0, 15, 30, or 60 min. (B) 3T3-L1 adipocytes were untreated (Ctrl), or treated with 1 ng/ml TNFα or 4 mM MβCD, together without or with 10 μM BMS-345541 for 30 min. Cellular proteins were solubilized and subjected to SDS-PAGE and Western blot analysis with the indicated antibodies. Representative immunoblots from three independent experiments are shown. (C, D) 3T3-L1 adipocytes were untreated (Ctrl), or treated with 1 ng/ml TNFα or 4 mM MβCD, together without or with 10 μM BMS-345541 for 4 h. The MCP-1 released into the media (C) and the MCP-1 protein in cell lysate (D) were determined. Each point represents the mean ± S.E. of three independent experiments. Asterisks denote significant differences (**p<0.01). (E) 3T3-L1 adipocytes were transfected with non-targeting luciferase siRNA (-) or siRNA against IKKβ (+). Protein levels of IKKβ were determined by Western blotting. (F) 3T3-L1 adipocytes were transfected with luciferase (Luc) or IKKβ siRNA. 48 h post transfection, cells were untreated (Ctrl) or treated with 4 mM MβCD for 4h. MCP-1 released into the media was determined by ELISA. Each point represents the mean ± S.E. of four independent experiments. Asterisks denote significant differences (*p<0.05, **p<0.01, ***p<0.001).

Activation of the transcription factor NF-κB can also increase MCP-1 expression [15, 16, 28, 31–33]. Therefore, we tested whether NF-κB is involved in MβCD-induced MCP-1 expression and secretion. Activation of NF-κB is usually followed by the phosphorylation and degradation of its inhibitor protein, inhibitor to I kappa B α (IκBα). To test whether MβCD treatment might activate the NF-κB pathway in adipocytes, differentiated adipocytes were treated with TNFα or MβCD for 0, 15, 30, or 60 min, and the phosphorylation and protein levels of IκBα were determined. As shown in Fig 4A, TNFα treatment induced IκBα phosphorylation and degradation as early as 15 min, suggesting the NF-κB signaling pathway is activated. MβCD treatment also induced IκBα phosphorylation and degradation, but were less potent compared with TNFα treatment. We also examined the cellular localization of the p65 subunit of NF-κB, which localizes into the nucleus after being activated. While the p65 remained in the cytoplasm of the untreated Ctrl cells, TNFα or MβCD treatment induced nuclear localization in adipocytes (S6 Fig). Moreover, MβCD treatment also increased the mRNA levels of other NF-κB-dependent genes (S7 Fig). These results demonstrate that MβCD treatment activates the NF-κB pathway in adipocytes.

To test whether activation of NF-κB is involved in MβCD-induced MCP-1 secretion, we used BMS-345541, the inhibitor of IκB kinase (IKK) [28, 34]. IKK is the upstream kinase that phosphorylates IκBα and leads to degradation of IκBα and activation of NF-κB. BMS-345541 treatment abolished TNFα- (Fig 4B, left panel) or MβCD-induced (Fig 4B, right panel) IκBα phosphorylation and degradation in 3T3-L1 adipocytes, consistent with its suppression of NF-κB activation. Additionally, BMS-345541 treatment not only reduced basal MCP-1 secretion, but also greatly decreased MβCD- or TNFα-induced MCP-1 secretion in adipocytes (Fig 4C). Consistent with the ELISA results, BMS-345541 treatment also reduced the MCP-1 protein increase induced by MβCD or TNFα treatment (Fig 4D). To confirm the results from pharmacological inhibition, we knocked down IKKβ (Fig 4E), the cellular target of BMS-345541. As shown in Fig 4F, depletion of IKKβ reduced MβCD-induced MCP-1 secretion. These data suggest that MβCD treatment activates the NF-κB pathway to increase MCP-1 biosynthesis and secretion.

### Filipin Treatment also Induces MCP-1 Expression and Secretion in 3T3-L1 Adipocytes without Affecting Cholesterol Levels

**Fig 5:**
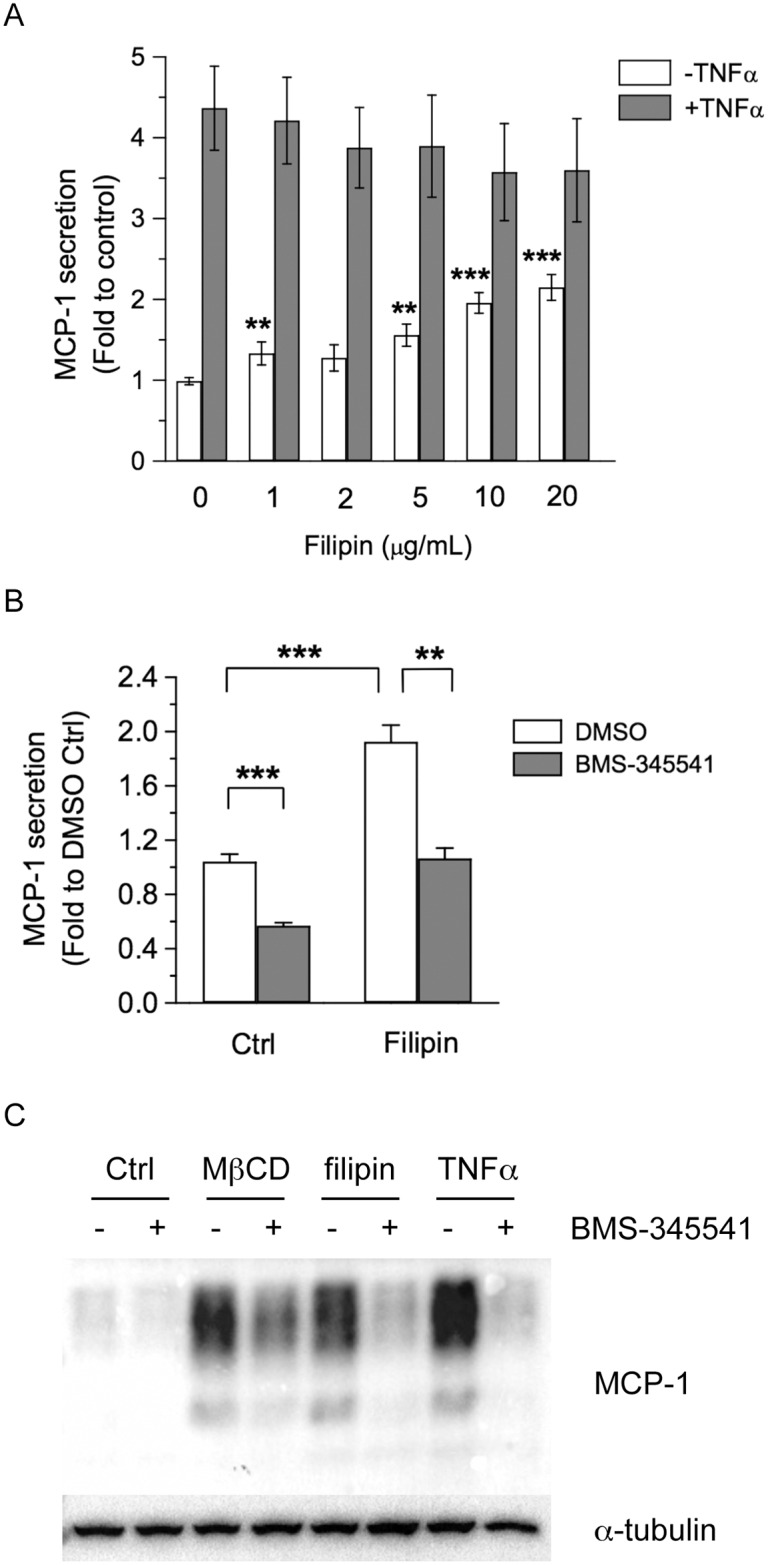
Filipin treatment increases MCP-1 secretion and protein expression. (A) 3T3-L1 adipocytes were treated with increasing doses of filipin (0, 1, 2, 5, 10, 20 μg/ml), together without or with 1 ng/ml TNFα for 4 h. MCP-1 released into the media was determined. Each point represents the mean ± S.E. of three independent experiments. Asterisks denote significant difference compared with untreated value of 0 μg/ml group (**p<0.01, ***p<0.001). (B) 3T3-L1 adipocytes were treated with vehicle (Ctrl) or 10 μg/ml filipin, together with DMSO or 10 μM BMS-345541 for 4 h. MCP-1 secretion into the media was determined. Each point represents the mean ± S.E. of three independent experiments. Asterisks denote significant difference (**p<0.01, ***p<0.001). (C) 3T3-L1 adipocytes were treated vehicle (Ctrl), 4 mM MβCD, 10 μg/ml filipin, or 1 ng/ml TNFα, together without or with 10 μM BMS-345541 for 4 h. Cellular proteins were solubilized and subjected to SDS-PAGE and Western blot analysis with the indicated antibodies. Representative immunoblots from three independent experiments are shown.

MβCD treatment depleted cholesterol and increased MCP-1 expression and secretion in adipocytes. Depletion of membrane cholesterol may lead to the disruption of lipid rafts, which are the microdomains of plasma membrane enriched in cholesterol and sphingolipids, as well as caveolae, a subset of lipid raft domains consisting of structure protein caveolin [11, 35]. To determine if another cholesterol-binding reagent would also induced MCP-1 similarly to MβCD, we treated 3T3-L1 adipocytes with the polyene antibiotic filipin, which can also bind and sequester membrane cholesterol [36]. We performed a dose response experiment of filipin treatment. As shown in Fig 5A, filipin treatment induced a dose-dependent increase of the MCP-1 secretion in 3T3-L1 adipocytes. Similarly to the results of the MβCD treatment, filipin treatment did not further increase TNFα-induced MCP-1 secretion (Fig 5A).

We also examined if NF-κB activation was involved in filipin-induced MCP-1 secretion. Treatment with BMS-345541 reduced basal and filipin-induced MCP-1 secretion, suggesting the involvement of NF-κB activation in filipin-induced MCP-1 secretion (Fig 5B). Filipin treatment by itself increased MCP-1 protein expression, whereas BMS-345541 treatment greatly reduced filipin-induced MCP-1 protein levels (Fig 5C). These results confirmed that filipin treatment, which also disrupts the integrity of lipid rafts like MβCD treatment, increases MCP-1 expression and secretion in adipocytes.

**Fig 6:**
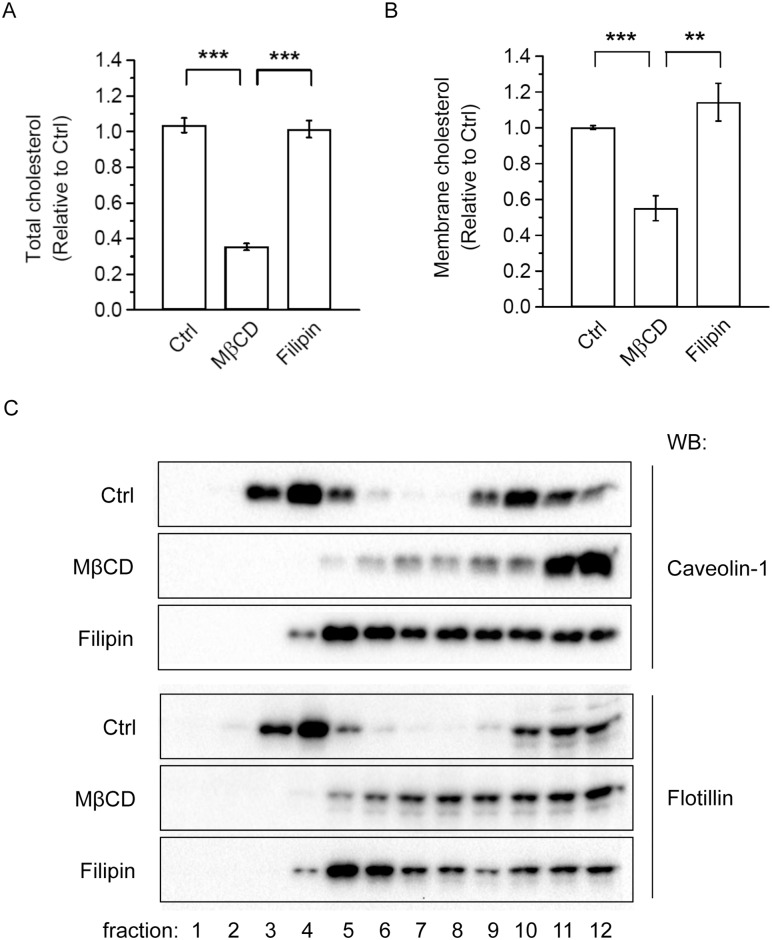
Effects of MβCD and filipin treatment on cholesterol levels and the membrane distribution of lipid raft proteins in adipocytes. (A) 3T3-L1 adipocytes were treated with vehicle (Ctrl), 4 mM MβCD, or 10 μg/ml filipin for 4 h. Cellular lysate was collected and cholesterol levels were determined as described in the Methods (B). 3T3-L1 adipocytes were treated with vehicle (Ctrl), 4 mM MβCD, or 10 μg/ml filipin for 4 h. The membrane fraction was collected after fractionation and cholesterol levels were determined. Each point represents the mean ± S.E. of three independent experiments. Asterisks denote significant differences (**p<0.01, ***p<0.001). (C) 3T3-L1 adipocytes were untreated (Ctrl) or treated with 4 mM MβCD or 10 μg/ml filipin for 4 h. Cells were lysed with 1% Triton X-100 at 4°C and subjected to sucrose density-gradient ultracentrifugation as described in Methods. Representative immunoblots from two independent experiments are shown.

MβCD is known to be a cholesterol acceptor that depletes cellular and membrane cholesterol levels. As shown in Fig 6A, 4 h MβCD treatment reduced the total cholesterol level in the adipocytes, consistent with its function as a cholesterol acceptor. In contrast, filipin treatment did not affect cholesterol levels (Fig 6A). To determine how MβCD or filipin treatment affected the membrane cholesterol level, we fractionated MβCD- or filipin- treated 3T3-L1 adipocyte cell lysates to obtain the membrane fraction. As expected, the 4 h MβCD treatment reduced membrane cholesterol to 50% of the Ctrl cells (Fig 6B). In contrast, filipin treatment did not affect membrane cholesterol (Fig 6B), which is consistent with its function to sequester membrane cholesterol without depleting it [36, 37]. Thus, MβCD treatment, which depletes cellular and membrane cholesterol, may increase MCP-1 expression and secretion and activate ERK and NF-κB by disrupting lipid raft integrity or function. To confirm this, we performed lipid raft fractionation assay. 3T3-L1 adipocytes were pretreated with vehicle (Ctrl), 4 mM MβCD, or 10 μg/ml filipin, and cell lysates were subjected to sucrose density-gradient ultracentrifugation. As shown in Fig 6C, distribution of lipid raft marker proteins (caveolin-1 and flotillin) was altered after treatment with MβCD or filipin, consistent with a disturbance of lipid raft integrity in adipocytes.

## Discussion

In this study, we used MβCD treatment to acutely deplete cholesterol to determine how MCP-1 secretion in 3T3-L1 adipocytes would be affected. Depletion of cholesterol induced increases in mRNA, protein, and secretion of MCP-1 in adipocytes through the activation of NF-κB. Moreover, the increase in MCP-1 expression and secretion and proinflammatory signaling due to MβCD-induced cholesterol depletion may be a result of the disturbance of lipid raft integrity, since filipin treatment, which did not alter membrane or total cholesterol levels, also increased MCP-1 expression and secretion. Filipin can insert into the cell membrane and disturb the function of lipid rafts by sequestering rather than extracting cholesterol [36, 37]. Additionally, we confirmed that MβCD and filipin treatment disturbed the integrity of lipid rafts in adipocytes (Fig 6C). Because cholesterol redistribution was observed in hypertrophied adipocytes [1–3, 5–7], the depletion of membrane cholesterol in obese adipocytes may result in a disturbance of the lipid raft function, leading to elevated MCP-1 expression and secretion and activation of proinflammatory signaling pathways such as ERK and NF-κB.

Lipid rafts have been shown to be involved in many cellular functions, including signaling [8, 38], exocytosis [39], and endocytosis [9]. However, the role of lipid rafts in modulating adipocyte function is not fully understood. Lipid rafts are necessary for the proper compartmentalization of insulin signaling. A portion of the insulin receptor is localized to caveolae, a subset of lipid rafts [40]. Intact lipid rafts are required for insulin-stimulated tyrosine phosphorylation of Cbl and the activation of small GTP binding protein TC10 in adipocytes. Disruption of lipid rafts inhibits insulin-stimulated TC10 activation and subsequent GLUT4 translocation [41–43]. Thus, it has been suggested that insulin resistance may be caused in part by the disruption of caveolae/lipid rafts that leads to altered localization of insulin receptor and disassembly of insulin signaling molecules [44, 45]. However, in addition to these effects, our findings suggest that the disturbance of lipid raft function may also activate proinflammatory signaling pathways (such as ERK and NF-κB) and increase MCP-1 production in adipocytes. Interestingly, both of these changes are known to contribute to insulin resistance in obesity [46].

Depending on the cell types and signaling pathways, disruption of lipid rafts may either activate or attenuate signaling events. Lipopolysaccharide (LPS)-stimulated signaling molecules are localized to and assemble in lipid rafts of human blood cells. Disruption of lipid rafts attenuates LPS-stimulation and subsequent TNFα secretion [47]. MβCD treatment disrupts cocaine-induced lipid raft-localization of sigma receptor and attenuates MCP-1 secretion in microglia [48]. However, disruption of lipid rafts may release the trapped signaling molecules from the rafts, leading to the activation of a signaling event. For example, the protein tyrosine kinase LCK is released from rafts after cholesterol depletion to induce tyrosine phosphorylation of multiple signaling molecules that activate the Ras-ERK pathway in T lymphocytes [49]. Disruption of membrane cholesterol results in activation of NF-κB in immature B cells, whereas mature B cells are insensitive to the same treatment [50]. In adipocytes, 30 h MβCD treatment attenuates palmitate-induced MCP-1 expression [51]. Apolipoprotein A-I, known to mediate cholesterol efflux through ATP binding cassette transporter A1, attenuates LPS-induced activation of MAPKs and NF-κB, as well as MCP-1 production in adipocytes [52]. Interestingly, both palmitate and LPS induce signal transduction via toll-like receptor 4 (TLR4) [53–55], a receptor known to localize to lipid rafts [54–56]. We found that cholesterol depletion did not inhibit TNFα-induced ERK phosphorylation (Fig 3A) and MCP-1 production (Figs 1B and 2D–2F) in adipocytes. Thus, cholesterol depletion or lipid raft disruption may attenuate signaling events mediated by TLR4, but not others (such as TNFα receptors) in adipocytes. Moreover, our results showed that cholesterol depletion by itself, which caused lipid raft disruption, could increase basal proinflammatory signaling and MCP-1 production in adipocytes.

We also examined if knockdown of caveolin-1, the structure protein of caveolae, might induce MCP-1 secretion in adipocytes as induced by MβCD treatment. However, depletion of caveolin-1 did not affect basal or TNFα-induced MCP-1 secretion in adipocytes (S8 Fig). Given that caveolae represent only a subgroup of lipid microdomains, disruption of caveolae may not affect the function of non-caveolar lipid rafts. Moreover, it has been reported that caveolae and non-caveolar lipid rafts may have distinct functions in adipocytes [57]. Alternatively, residual caveolin-1/caveolae after knockdown may be enough for cellular function. Adipocytes from caveolin-1 knockout mice may help to address this question.

An early report showing that during adipocyte differentiation, a 4-day inhibition of sterol synthesis results in elevated expression of TNFα and IL-6 [7]. The authors attribute the up-regulation of proinflammatory cytokines to the depletion of cholesterol in differentiating adipocytes. However, it is possible that chronic depletion of cholesterol by inhibiting sterol synthesis during adipocyte differentiation may also lead to disruption of lipid raft function. This effect could cause the elevated expression of these proinflammatory cytokines found in the study.

The discrepancy between pharmacological inhibition and MEK1/2 knockdown remains to be elucidated. Although modulation of cholesterol levels by MβCD or WSCL treatment regulated ERK phosphorylation (S4 Fig), knockdown of MEK1/2, the upstream kinases of ERK1/2, did not affect MβCD-induced MCP-1 secretion (Fig 3G). Moreover, basal and MβCD-induced ERK1/2 phosphorylation were elevated in MEK1/2-depleted adipocytes (Fig 3F). The duration of pharmacological inhibition (4 h including the pretreatment) was usually shorter than the knockdown experiment (last from 24–72 h). Compensated mechanism or elevation of other signaling pathways may occur after chronic depletion of MEK1/2. Alternatively, MEK-independent mechanisms have been reported for chronic activation of ERK1/2, such as PI3K/Akt pathways [58, 59]. We did not observe activation of Akt, the downstream substrate of PI3K, in MβCD-treated adipocytes (data not shown). Treatment with selective PI3K inhibitor LY294002 only moderately reduced MβCD-induced MCP-1 secretion. Further experiments are required to elucidate these MEK-independent mechanisms.

The mechanism that leads from disruption of lipid rafts to activation of ERK or NF-κB and increased MCP-1 production in adipocytes remains to be determined. It has been shown that intact lipid rafts are required for long-chain fatty acid uptake in adipocytes [60, 61]. Depletion of membrane cholesterol, which disrupts the lipid raft association of the fatty acid transporter FAT/CD36, inhibits fatty acid uptake [61]. Moreover, free fatty acids have been shown to induce secretion of chemokines including MCP-1 in 3T3-L1 adipocytes [16]. We did observe elevated levels of non-esterified fatty acid (NEFA), but not glycerol, in culture medium of 3T3-L1 adipocytes during longer treatment (24 h) with MβCD (S9 Fig). However, shorter incubation (such as 4 h or less in most of our experiments) with MβCD did not cause measureable fatty acids in the medium. These observations eliminate the elevation of fatty acids in medium as a cause for MβCD-induced MCP-1 secretion or activation of NF-κB in our experimental setup.

With the high affinity for cholesterol, cyclodextrins have been used to manipulate cellular cholesterol levels. Cyclodextrins, particularly β-cyclodextrins (β-CDs) were widely used as pharmaceutical excipients of poorly water-soluble drugs [62]. More recently, the use of 2-hydroxypropyl-β-cyclodextrin (HPB-CD) in treating patients with Niemann-Pick type C disease, a neurodegenerative disease with massive accumulation of cholesterol in the endosomes/lysosomes [63], highlights the potential effects of cyclodextrins in human physiology. Thus, a better understanding of the cellular mechanisms of cyclodextrins may allow for better therapeutic strategies in the future.

## Supporting Information

S1 Fig Treatment with cholesterol synthesis inhibitors does not alter cholesterol levels in adipocytes. 3T3-L1 adipocytes were treated with vehicle (Ctrl), 10 μg/ml mevastatin, or 10 μg/ml U18666A in serum free DMEM for 24 h. Cell lysate was collected and cholesterol levels were determined. Each point represents the mean ± S.E. of three independent experiments.
(TIF)

S2 Fig Dose effects of MβCD on cell viability. 3T3-L1 adipocytes were treated with increasing MβCD doses (0, 1.875, 2.5, 3.75, 5, 7.5, or 10 mM) for 24 h. Cell viability was determined by the MTT assay. Each point represents the mean ± S.E. of three independent experiments. Asterisks denote significant difference compared with 0 mM control (**p<0.01, ***p<0.001).
(TIF)

S3 Fig Cycloheximide treatment abolishes basal, MβCD, or TNFα-induced MCP-1 secretion. 3T3-L1 adipocytes were untreated (Ctrl), or treated with 4 mM MβCD or 1 ng/ml TNFα, together with DMSO or 50 μg/ml cycloheximide for 4 h. MCP-1 release in the medium was determined by ELISA. Each point represents the mean ± S.E. of three independent experiments. Asterisks denote significant difference (***p<0.001) compared with DMSO treatment in each group.
(TIF)

S4 Fig Modulations of cholesterol levels regulate basal and TNFα-induced ERK phosphorylation. 3T3-L1 adipocytes were untreated (Ctrl), or pretreated with 4 mM MβCD, 250 μg/ml WSCL, or both for 4 h, and then treated with increasing TNFα doses (0, 0.25, 1 ng/ml) for 30 min. Cellular proteins were solubilized and subjected to SDS-PAGE and Western blot analysis with the indicated antibodies. Representative immunoblots from three independent experiments are shown.
(TIF)

S5 Fig Effects of JNK and p38 inhibitors on MβCD-induced MCP-1 expression and secretion. (A, B) 3T3-L1 adipocytes were untreated (Ctrl), or treated with 4 mM MβCD or 1 ng/ml TNFα, together without (DMSO) or with 10 μM SP600125 for 4 h. (A) Cellular proteins were solubilized and subjected to SDS-PAGE and Western blot analysis with the indicated antibodies. Representative immunoblots from three independent experiments are shown. (B) MCP-1 secretion in medium was determined by ELISA. Each point represents the mean ± S.E. of four independent experiments. Asterisks denote significant difference (*p<0.05, **p<0.01). NS, not significant. (C, D) 3T3-L1 adipocytes were untreated (Ctrl), or treated with 4 mM MβCD or 1 ng/ml TNFα, together without (DMSO) or with 10 μM SB203580 for 4 h. (C) Cellular proteins were solubilized and subjected to SDS-PAGE and Western blot analysis with the indicated antibodies. Representative immunoblots from three independent experiments are shown. (D) MCP-1 secretion in medium was determined by ELISA. Each point represents the mean ± S.E. of five independent experiments. Asterisks denote significant difference (*p<0.05, ***p<0.001). NS, not significant.
(TIF)

S6 Fig MβCD or TNFα treatment stimulates nuclear localization of p65 NF-κB. 3T3-L1 adipocytes were untreated (Ctrl), or treated with 4 mM MβCD or 1 ng/ml TNFα for 30 min. NF-κB localization was stained with anti-p65 NF-κB antibody (red). Nuclei were stained with DAPI (blue). Scale bar = 10 μm.
(TIF)

S7 Fig MβCD treatment increased mRNA levels of NF-κB-dependent genes. 3T3-L1 adipocytes were untreated (Ctrl) or treated with 4 mM MβCD for 0.5, 1, 2, or 4 h. mRNA levels of IL-6 (A) and IκBα (B) were determined by qPCR. Each point represents the mean ± S.E. of at least three independent experiments. Asterisks denote significant differences (*p<0.05, **p<0.01).
(TIF)

S8 Fig Knockdown of caveolin-1 did not affect basal or TNFα-induced MCP-1 secretion. (A) 3T3-L1 adipocytes were transfected with non-targeting luciferase siRNA (-) or siRNA against caveolin-1 (+). Protein levels of caveolin-1 (Cav-1) were determined by Western blotting. (B) 3T3-L1 adipocytes were transfected with luciferase (Luc) or caveolin-1 (Cav-1) siRNA. 48 h post transfection, cells were untreated (Ctrl) or treated with 1 ng/ml TNFα for 4h. MCP-1 released into the media was determined by ELISA. Each point represents the mean ± S.E. of three independent experiments. Asterisks denote significant differences (*p<0.05, compared with Ctrl value).
(TIF)

S9 Fig Chronic treatment with MβCD increases NEFA release in adipocytes. 3T3-L1 adipocytes were untreated (Ctrl), or treated with 4 mM MβCD, 250 μg/ml WSCL, or both for 24 h. Glycerol (A) or NEFA (B) release into the media and protein concentrations of cell lysate were determined. Each point represents the mean ± S.E. of at least three independent experiments. Asterisks denote significant differences (*p<0.05, **p<0.01).
(TIF)

S1 File Supporting Information Methods. (DOCX)

## References

[1] Yu BL, Zhao SP, Hu JR. Cholesterol imbalance in adipocytes: a possible mechanism of adipocytes dysfunction in obesity. Obes Rev. 2010;11(8):560–7. Epub 2009/12/23. 10.1111/j.1467-789X.2009.00699.x 20025694

[2] Aguilar D, Fernandez ML. Hypercholesterolemia induces adipose dysfunction in conditions of obesity and nonobesity. Adv Nutr. 2014;5(5):497–502. 10.3945/an.114.005934 25469381 PMC4188221

[3] Dugail I, Le Lay S, Varret M, Le Liepvre X, Dagher G, Ferre P. New insights into how adipocytes sense their triglyceride stores. Is cholesterol a signal? Horm Metab Res. 2003;35(4):204–10. Epub 2003/06/05. 10.1055/s-2003-39475 12778362

[4] Schreibman PH, Dell RB. Human adipocyte cholesterol. Concentration, localization, synthesis, and turnover. J Clin Invest. 1975;55(5):986–93. 10.1172/JCI108028 1123433 PMC301844

[5] Farkas J, Angel A, Avigan MI. Studies on the compartmentation of lipid in adipose cells. II. Cholesterol accumulation and distribution in adipose tissue components. J Lipid Res. 1973;14(3):344–56. 9704080

[6] Guerre-Millo M, Guesnet P, Guichard C, Durand G, Lavau M. Alteration in membrane lipid order and composition in metabolically hyperactive fatty rat adipocytes. Lipids. 1994;29(3):205–9. 8170290 10.1007/BF02536730

[7] Le Lay S, Krief S, Farnier C, Lefrere I, Le Liepvre X, Bazin R, et al Cholesterol, a cell size-dependent signal that regulates glucose metabolism and gene expression in adipocytes. J Biol Chem. 2001;276(20):16904–10. Epub 2001/03/30. 10.1074/jbc.M010955200 11278795

[8] Lemaire-Ewing S, Lagrost L, Neel D. Lipid rafts: a signalling platform linking lipoprotein metabolism to atherogenesis. Atherosclerosis. 2012;221(2):303–10. Epub 2011/11/11. 10.1016/j.atherosclerosis.2011.10.016 22071358

[9] Lajoie P, Nabi IR. Lipid rafts, caveolae, and their endocytosis. Int Rev Cell Mol Biol. 2010;282:135–63. Epub 2010/07/16. 10.1016/S1937-6448(10)82003-9 20630468

[10] Surma MA, Klose C, Simons K. Lipid-dependent protein sorting at the trans-Golgi network. Biochimica et biophysica acta. 2012;1821(8):1059–67. Epub 2012/01/11. 10.1016/j.bbalip.2011.12.008 22230596

[11] Breen MR, Camps M, Carvalho-Simoes F, Zorzano A, Pilch PF. Cholesterol depletion in adipocytes causes caveolae collapse concomitant with proteosomal degradation of cavin-2 in a switch-like fashion. PLoS One. 2012;7(4):e34516 Epub 2012/04/12. 10.1371/journal.pone.0034516 22493697 PMC3321009

[12] Chamberlain LH, Gould GW. The vesicle- and target-SNARE proteins that mediate Glut4 vesicle fusion are localized in detergent-insoluble lipid rafts present on distinct intracellular membranes. J Biol Chem. 2002;277(51):49750–4. 10.1074/jbc.M206936200 12376543

[13] Chiang SH, Baumann CA, Kanzaki M, Thurmond DC, Watson RT, Neudauer CL, et al Insulin-stimulated GLUT4 translocation requires the CAP-dependent activation of TC10. Nature. 2001;410(6831):944–8. 10.1038/35073608 11309621

[14] Kanda H, Tateya S, Tamori Y, Kotani K, Hiasa K, Kitazawa R, et al MCP-1 contributes to macrophage infiltration into adipose tissue, insulin resistance, and hepatic steatosis in obesity. J Clin Invest. 2006;116(6):1494–505. 10.1172/JCI26498 16691291 PMC1459069

[15] Wuyts WA, Vanaudenaerde BM, Dupont LJ, Demedts MG, Verleden GM. Involvement of p38 MAPK, JNK, p42/p44 ERK and NF-kappaB in IL-1beta-induced chemokine release in human airway smooth muscle cells. Respiratory medicine. 2003;97(7):811–7. 12854631 10.1016/s0954-6111(03)00036-2

[16] Jiao P, Chen Q, Shah S, Du J, Tao B, Tzameli I, et al Obesity-related upregulation of monocyte chemotactic factors in adipocytes: involvement of nuclear factor-kappaB and c-Jun NH2-terminal kinase pathways. Diabetes. 2009;58(1):104–15. 10.2337/db07-1344 18835938 PMC2606857

[17] Sell H, Eckel J. Monocyte chemotactic protein-1 and its role in insulin resistance. Curr Opin Lipidol. 2007;18(3):258–62. 10.1097/MOL.0b013e3281338546 17495598

[18] Niu J, Kolattukudy PE. Role of MCP-1 in cardiovascular disease: molecular mechanisms and clinical implications. Clin Sci (Lond). 2009;117(3):95–109. 19566488 10.1042/CS20080581

[19] Christian AE, Haynes MP, Phillips MC, Rothblat GH. Use of cyclodextrins for manipulating cellular cholesterol content. J Lipid Res. 1997;38(11):2264–72. 9392424

[20] Lu JC, Chang YT, Wang CT, Lin YC, Lin CK, Wu ZS. Trichostatin A Modulates Thiazolidinedione-Mediated Suppression of Tumor Necrosis Factor alpha-Induced Lipolysis in 3T3-L1 Adipocytes. PLoS One. 2013;8(8):e71517 Epub 2013/08/21. 10.1371/journal.pone.0071517 23951179 PMC3739734

[21] Gonzalez-Munoz E, Lopez-Iglesias C, Calvo M, Palacin M, Zorzano A, Camps M. Caveolin-1 loss of function accelerates glucose transporter 4 and insulin receptor degradation in 3T3-L1 adipocytes. Endocrinology. 2009;150(8):3493–502. Epub 2009/05/02. 10.1210/en.2008-1520 19406948

[22] Brown DA, Rose JK. Sorting of GPI-anchored proteins to glycolipid-enriched membrane subdomains during transport to the apical cell surface. Cell. 1992;68(3):533–44. 1531449 10.1016/0092-8674(92)90189-j

[23] Cenedella RJ. Cholesterol synthesis inhibitor U18666A and the role of sterol metabolism and trafficking in numerous pathophysiological processes. Lipids. 2009;44(6):477–87. Epub 2009/05/15. 10.1007/s11745-009-3305-7 19440746

[24] Han J, Hajjar DP, Tauras JM, Nicholson AC. Cellular cholesterol regulates expression of the macrophage type B scavenger receptor, CD36. J Lipid Res. 1999;40(5):830–8. Epub 1999/05/01. 10224152

[25] Bogan JS, Xu Y, Hao M. Cholesterol accumulation increases insulin granule size and impairs membrane trafficking. Traffic. 2012;13(11):1466–80. Epub 2012/08/15. 10.1111/j.1600-0854.2012.01407.x 22889194 PMC3465494

[26] Ito A, Suganami T, Miyamoto Y, Yoshimasa Y, Takeya M, Kamei Y, et al Role of MAPK phosphatase-1 in the induction of monocyte chemoattractant protein-1 during the course of adipocyte hypertrophy. J Biol Chem. 2007;282(35):25445–52. 10.1074/jbc.M701549200 17611196

[27] Ahmed RA, Murao K, Imachi H, Yoshida K, Dobashi H, Hosomi N, et al c-Jun N-terminal kinases inhibitor suppresses the TNF-alpha induced MCP-1 expression in human umbilical vein endothelial cells. Endocrine. 2009;35(2):184–8. 10.1007/s12020-008-9136-0 19107603

[28] Takahashi K, Yamaguchi S, Shimoyama T, Seki H, Miyokawa K, Katsuta H, et al JNK- and IkappaB-dependent pathways regulate MCP-1 but not adiponectin release from artificially hypertrophied 3T3-L1 adipocytes preloaded with palmitate in vitro. Am J Physiol Endocrinol Metab. 2008;294(5):E898–909. 10.1152/ajpendo.00131.2007 18303122

[29] Takahashi K, Miyokawa-Gorin K, Handa K, Kitahara A, Moriya R, Onuma H, et al Endogenous oxidative stress, but not ER stress, induces hypoxia-independent VEGF120 release through PI3K-dependent pathways in 3T3-L1 adipocytes. Obesity (Silver Spring). 2013;21(8):1625–34. 23670941 10.1002/oby.20206

[30] Yoshida T, Kakegawa J, Yamaguchi T, Hantani Y, Okajima N, Sakai T, et al Identification and characterization of a novel chemotype MEK inhibitor able to alter the phosphorylation state of MEK1/2. Oncotarget. 2012;3(12):1533–45. 10.18632/oncotarget.747 23237773 PMC3681492

[31] Ueda A, Okuda K, Ohno S, Shirai A, Igarashi T, Matsunaga K, et al NF-kappa B and Sp1 regulate transcription of the human monocyte chemoattractant protein-1 gene. J Immunol. 1994;153(5):2052–63. Epub 1994/09/01. 8051410

[32] Tsuchiya K, Yoshimoto T, Hirono Y, Tateno T, Sugiyama T, Hirata Y. Angiotensin II induces monocyte chemoattractant protein-1 expression via a nuclear factor-kappaB-dependent pathway in rat preadipocytes. Am J Physiol Endocrinol Metab. 2006;291(4):E771–8. 10.1152/ajpendo.00560.2005 16705055

[33] Yang X, Kume S, Tanaka Y, Isshiki K, Araki S, Chin-Kanasaki M, et al GW501516, a PPARdelta agonist, ameliorates tubulointerstitial inflammation in proteinuric kidney disease via inhibition of TAK1-NFkappaB pathway in mice. PLoS One. 2011;6(9):e25271 10.1371/journal.pone.0025271 21966476 PMC3178624

[34] Burke JR, Pattoli MA, Gregor KR, Brassil PJ, MacMaster JF, McIntyre KW, et al BMS-345541 is a highly selective inhibitor of I kappa B kinase that binds at an allosteric site of the enzyme and blocks NF-kappa B-dependent transcription in mice. J Biol Chem. 2003;278(3):1450–6. 10.1074/jbc.M209677200 12403772

[35] Marwali MR, Rey-Ladino J, Dreolini L, Shaw D, Takei F. Membrane cholesterol regulates LFA-1 function and lipid raft heterogeneity. Blood. 2003;102(1):215–22. 10.1182/blood-2002-10-3195 12637320

[36] Cuddy LK, Winick-Ng W, Rylett RJ. Regulation of the high-affinity choline transporter activity and trafficking by its association with cholesterol-rich lipid rafts. J Neurochem. 2014;128(5):725–40. 10.1111/jnc.12490 24127780

[37] Awasthi-Kalia M, Schnetkamp PP, Deans JP. Differential effects of filipin and methyl-beta-cyclodextrin on B cell receptor signaling. Biochem Biophys Res Commun. 2001;287(1):77–82. 10.1006/bbrc.2001.5536 11549256

[38] Lingwood D, Simons K. Lipid rafts as a membrane-organizing principle. Science. 2010;327(5961):46–50. Epub 2010/01/02. 10.1126/science.1174621 20044567

[39] Salaun C, James DJ, Chamberlain LH. Lipid rafts and the regulation of exocytosis. Traffic. 2004;5(4):255–64. Epub 2004/03/20. 10.1111/j.1600-0854.2004.0162.x 15030567 PMC2394575

[40] Kimura A, Mora S, Shigematsu S, Pessin JE, Saltiel AR. The insulin receptor catalyzes the tyrosine phosphorylation of caveolin-1. J Biol Chem. 2002;277(33):30153–8. 10.1074/jbc.M203375200 12036959

[41] Baumann CA, Ribon V, Kanzaki M, Thurmond DC, Mora S, Shigematsu S, et al CAP defines a second signalling pathway required for insulin-stimulated glucose transport. Nature. 2000;407(6801):202–7. 10.1038/35025089 11001060

[42] Chiang SH, Chang L, Saltiel AR. TC10 and insulin-stimulated glucose transport. Methods Enzymol. 2006;406:701–14. 10.1016/S0076-6879(06)06055-1 16472699

[43] Watson RT, Shigematsu S, Chiang SH, Mora S, Kanzaki M, Macara IG, et al Lipid raft microdomain compartmentalization of TC10 is required for insulin signaling and GLUT4 translocation. The Journal of cell biology. 2001;154(4):829–40. Epub 2001/08/15. 10.1083/jcb.200102078 11502760 PMC2196453

[44] Bickel PE. Lipid rafts and insulin signaling. Am J Physiol Endocrinol Metab. 2002;282(1):E1–E10. 11739076 10.1152/ajpendo.2002.282.1.E1

[45] Ikonen E, Vainio S. Lipid microdomains and insulin resistance: is there a connection? Sci STKE. 2005;2005(268):pe3 10.1126/stke.2682005pe3 15671480

[46] Tilg H, Moschen AR. Inflammatory mechanisms in the regulation of insulin resistance. Mol Med. 2008;14(3–4):222–31. 10.2119/2007-00119.Tilg 18235842 PMC2215762

[47] Triantafilou M, Miyake K, Golenbock DT, Triantafilou K. Mediators of innate immune recognition of bacteria concentrate in lipid rafts and facilitate lipopolysaccharide-induced cell activation. J Cell Sci. 2002;115(Pt 12):2603–11. 12045230 10.1242/jcs.115.12.2603

[48] Yao H, Yang Y, Kim KJ, Bethel-Brown C, Gong N, Funa K, et al Molecular mechanisms involving sigma receptor-mediated induction of MCP-1: implication for increased monocyte transmigration. Blood. 2010;115(23):4951–62. 10.1182/blood-2010-01-266221 20354174 PMC2890169

[49] Kabouridis PS, Janzen J, Magee AL, Ley SC. Cholesterol depletion disrupts lipid rafts and modulates the activity of multiple signaling pathways in T lymphocytes. Eur J Immunol. 2000;30(3):954–63. 10.1002/1521-4141(200003)30:3<954::AID-IMMU954>3.0.CO;2-Y 10741414

[50] Flemming JA, Perkins KH, Luus L, Ferguson AR, Corley RB. Disruption of membrane cholesterol stimulates MyD88-dependent NF-kappaB activation in immature B cells. Cell Immunol. 2004;229(1):68–77. 10.1016/j.cellimm.2004.06.004 15331330

[51] Umemoto T, Han CY, Mitra P, Averill MM, Tang C, Goodspeed L, et al Apolipoprotein AI and high-density lipoprotein have anti-inflammatory effects on adipocytes via cholesterol transporters: ATP-binding cassette A-1, ATP-binding cassette G-1, and scavenger receptor B-1. Circ Res. 2013;112(10):1345–54. 10.1161/CIRCRESAHA.111.300581 23501697 PMC3767575

[52] Sultana A, Cochran BJ, Tabet F, Patel M, Torres LC, Barter PJ, et al Inhibition of inflammatory signaling pathways in 3T3-L1 adipocytes by apolipoprotein A-I. FASEB J. 2016. 10.1096/fj.201500026R 26965683

[53] Han CY, Kargi AY, Omer M, Chan CK, Wabitsch M, O'Brien KD, et al Differential effect of saturated and unsaturated free fatty acids on the generation of monocyte adhesion and chemotactic factors by adipocytes: dissociation of adipocyte hypertrophy from inflammation. Diabetes. 2010;59(2):386–96. 10.2337/db09-0925 19934003 PMC2809975

[54] Cheng AM, Handa P, Tateya S, Schwartz J, Tang C, Mitra P, et al Apolipoprotein A-I attenuates palmitate-mediated NF-kappaB activation by reducing Toll-like receptor-4 recruitment into lipid rafts. PLoS One. 2012;7(3):e33917 10.1371/journal.pone.0033917 22479476 PMC3316516

[55] Shi H, Kokoeva MV, Inouye K, Tzameli I, Yin H, Flier JS. TLR4 links innate immunity and fatty acid-induced insulin resistance. J Clin Invest. 2006;116(11):3015–25. 10.1172/JCI28898 17053832 PMC1616196

[56] Olsson S, Sundler R. The role of lipid rafts in LPS-induced signaling in a macrophage cell line. Mol Immunol. 2006;43(6):607–12. 10.1016/j.molimm.2005.04.011 15904959

[57] Yao Y, Hong S, Zhou H, Yuan T, Zeng R, Liao K. The differential protein and lipid compositions of noncaveolar lipid microdomains and caveolae. Cell Res. 2009;19(4):497–506. 10.1038/cr.2009.27 19255590

[58] Grammer TC, Blenis J. Evidence for MEK-independent pathways regulating the prolonged activation of the ERK-MAP kinases. Oncogene. 1997;14(14):1635–42. 10.1038/sj.onc.1201000 9135064

[59] Aksamitiene E, Kholodenko BN, Kolch W, Hoek JB, Kiyatkin A. PI3K/Akt-sensitive MEK-independent compensatory circuit of ERK activation in ER-positive PI3K-mutant T47D breast cancer cells. Cell Signal. 2010;22(9):1369–78. 10.1016/j.cellsig.2010.05.006 20471474 PMC2893265

[60] Pohl J, Ring A, Korkmaz U, Ehehalt R, Stremmel W. FAT/CD36-mediated long-chain fatty acid uptake in adipocytes requires plasma membrane rafts. Mol Biol Cell. 2005;16(1):24–31. 10.1091/mbc.E04-07-0616 15496455 PMC539148

[61] Ehehalt R, Sparla R, Kulaksiz H, Herrmann T, Fullekrug J, Stremmel W. Uptake of long chain fatty acids is regulated by dynamic interaction of FAT/CD36 with cholesterol/sphingolipid enriched microdomains (lipid rafts). BMC Cell Biol. 2008;9:45 10.1186/1471-2121-9-45 18700980 PMC2533316

[62] Loftsson T, Jarho P, Masson M, Jarvinen T. Cyclodextrins in drug delivery. Expert Opin Drug Deliv. 2005;2(2):335–51. 10.1517/17425247.2.1.335 16296758

[63] Ottinger EA, Kao ML, Carrillo-Carrasco N, Yanjanin N, Shankar RK, Janssen M, et al Collaborative development of 2-hydroxypropyl-beta-cyclodextrin for the treatment of Niemann-Pick type C1 disease. Curr Top Med Chem. 2014;14(3):330–9. 24283970 10.2174/1568026613666131127160118 PMC4048128

