# Supplementary figures and images for: Disruption of Lipid Raft Function Increases Expression and Secretion of Monocyte Chemoattractant Protein-1 in 3T3-L1 Adipocytes
Source: PLoS One. 2016 Dec 28;11(12):e0169005. doi: 10.1371/journal.pone.0169005 (PMC5193455; doi:10.1371/journal.pone.0169005)

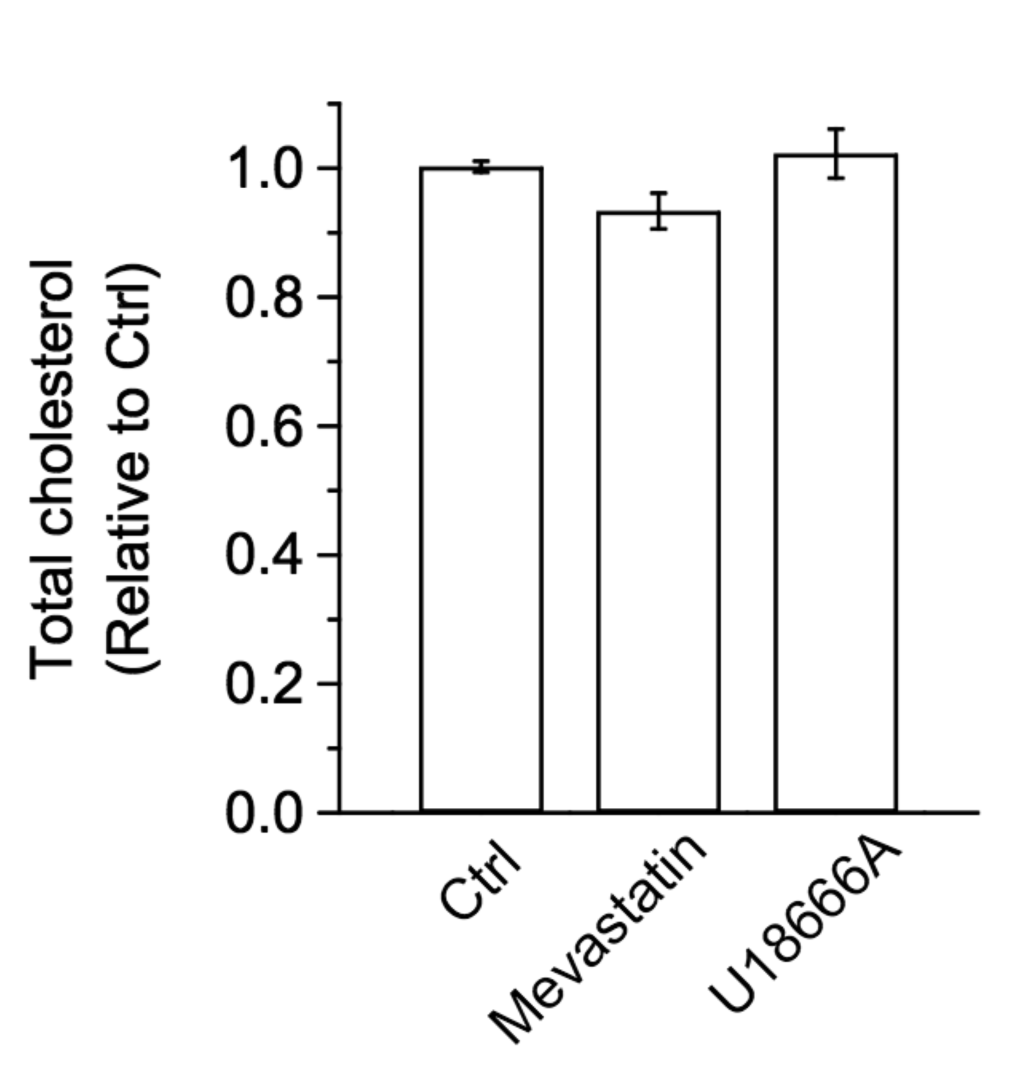

Supplement: S1 Fig — 3T3-L1 adipocytes were treated with vehicle (Ctrl), 10 μg/ml mevastatin, or 10 μg/ml U18666A in serum free DMEM for 24 h. Cell lysate was collected and cholesterol levels were determined. Each point represents the mean ± S.E. of three independent experiments. (TIF) [file pone.0169005.s001.tif]

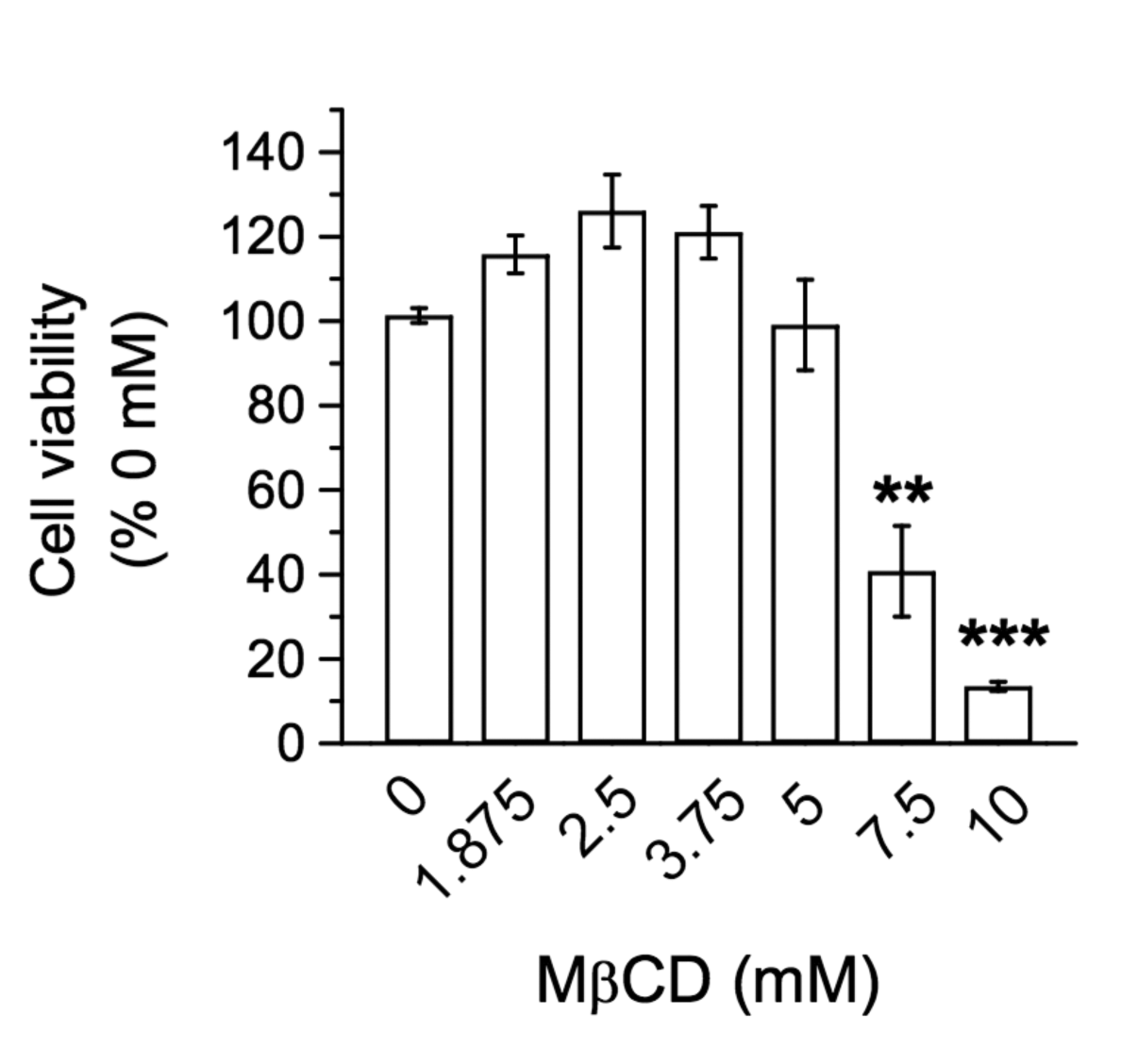

Supplement: S2 Fig — 3T3-L1 adipocytes were treated with increasing MβCD doses (0, 1.875, 2.5, 3.75, 5, 7.5, or 10 mM) for 24 h. Cell viability was determined by the MTT assay. Each point represents the mean ± S.E. of three independent experiments. Asterisks denote significant difference compared with 0 mM control (**p<0.01, ***p<0.001). (TIF) [file pone.0169005.s002.tif]

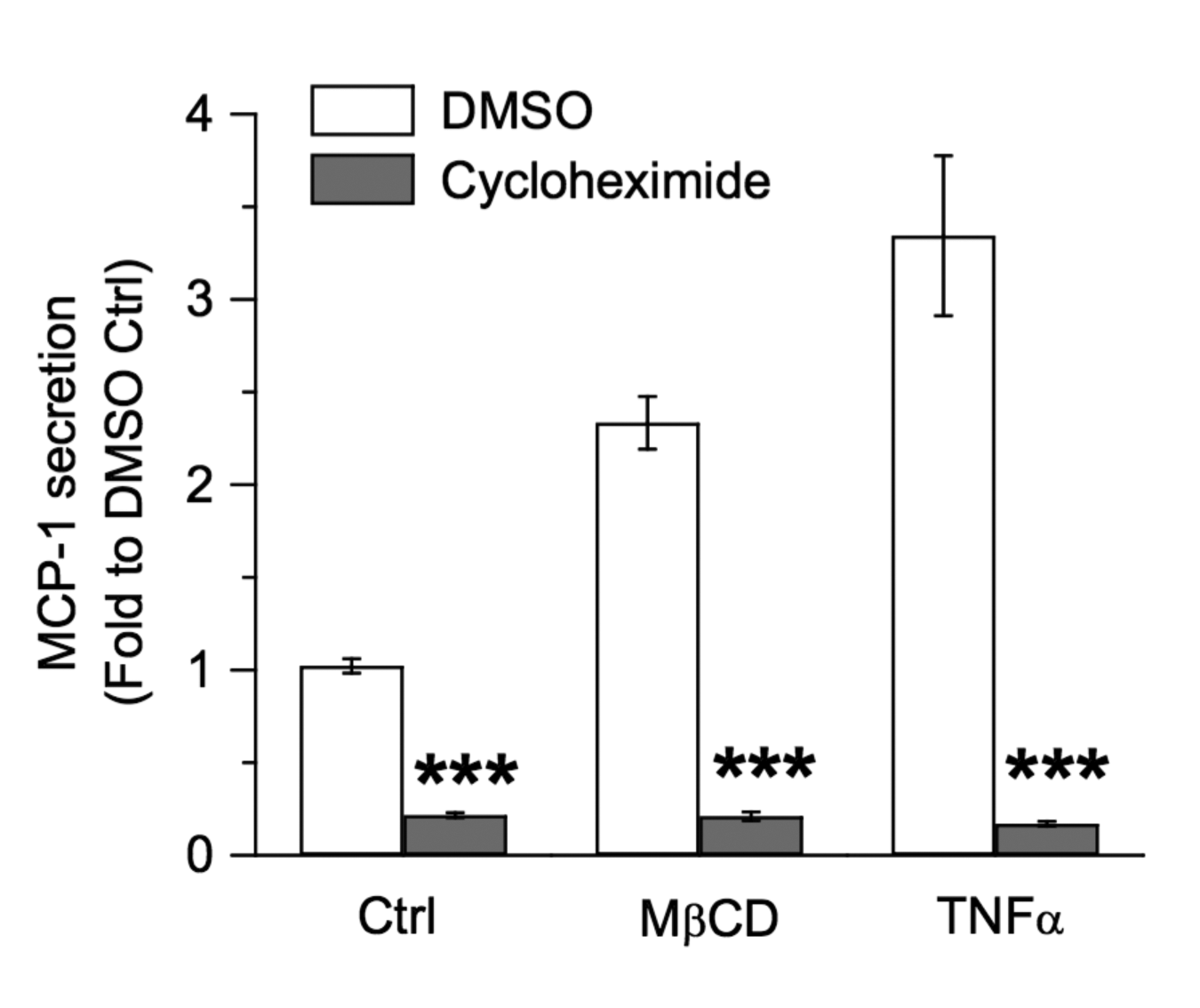

Supplement: S3 Fig — 3T3-L1 adipocytes were untreated (Ctrl), or treated with 4 mM MβCD or 1 ng/ml TNFα, together with DMSO or 50 μg/ml cycloheximide for 4 h. MCP-1 release in the medium was determined by ELISA. Each point represents the mean ± S.E. of three independent experiments. Asterisks denote significant difference (***p<0.001) compared with DMSO treatment in each group. (TIF) [file pone.0169005.s003.tif]

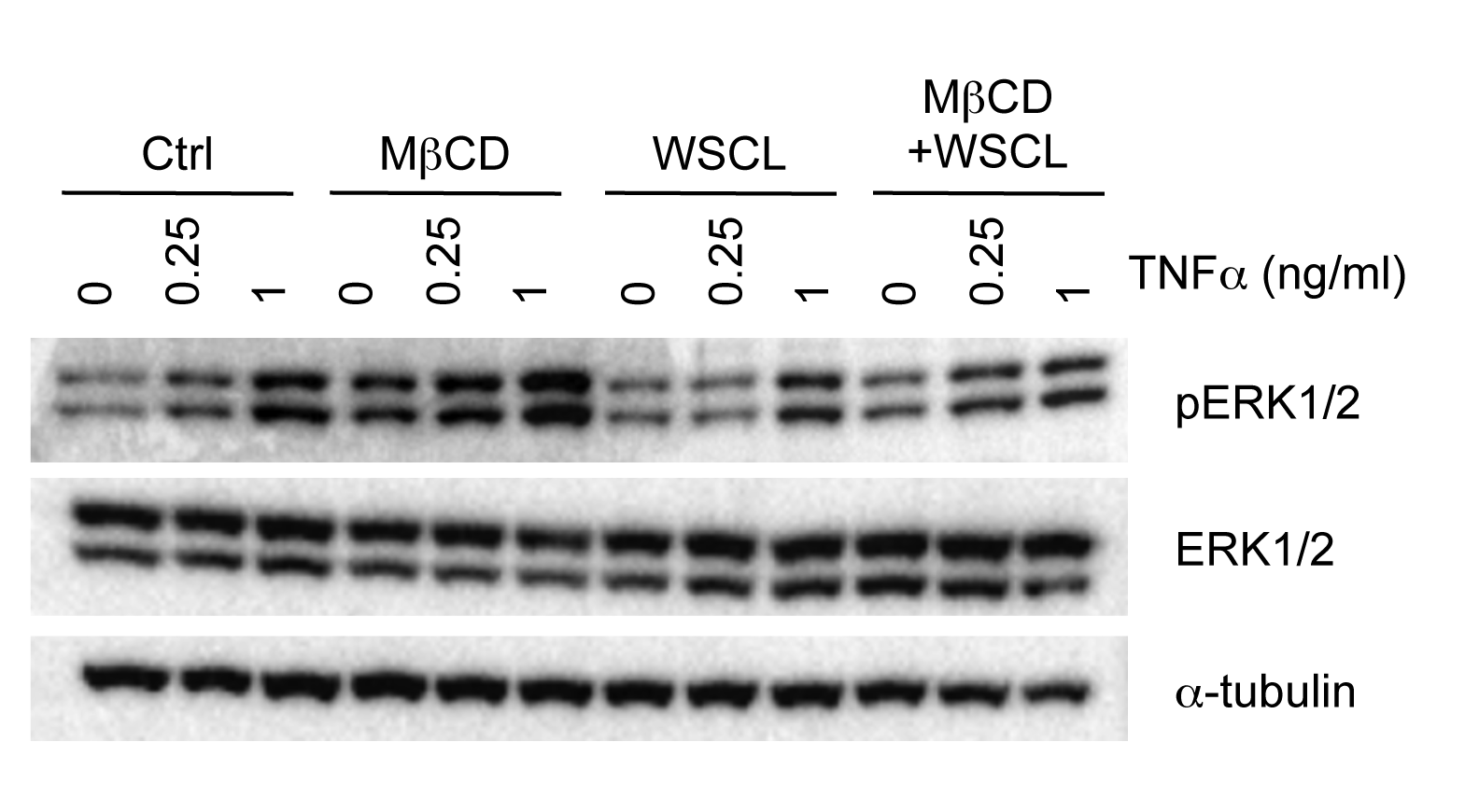

Supplement: S4 Fig — 3T3-L1 adipocytes were untreated (Ctrl), or pretreated with 4 mM MβCD, 250 μg/ml WSCL, or both for 4 h, and then treated with increasing TNFα doses (0, 0.25, 1 ng/ml) for 30 min. Cellular proteins were solubilized and subjected to SDS-PAGE and Western blot analysis with the indicated antibodies. Representative immunoblots from three independent experiments are shown. (TIF) [file pone.0169005.s004.tif]

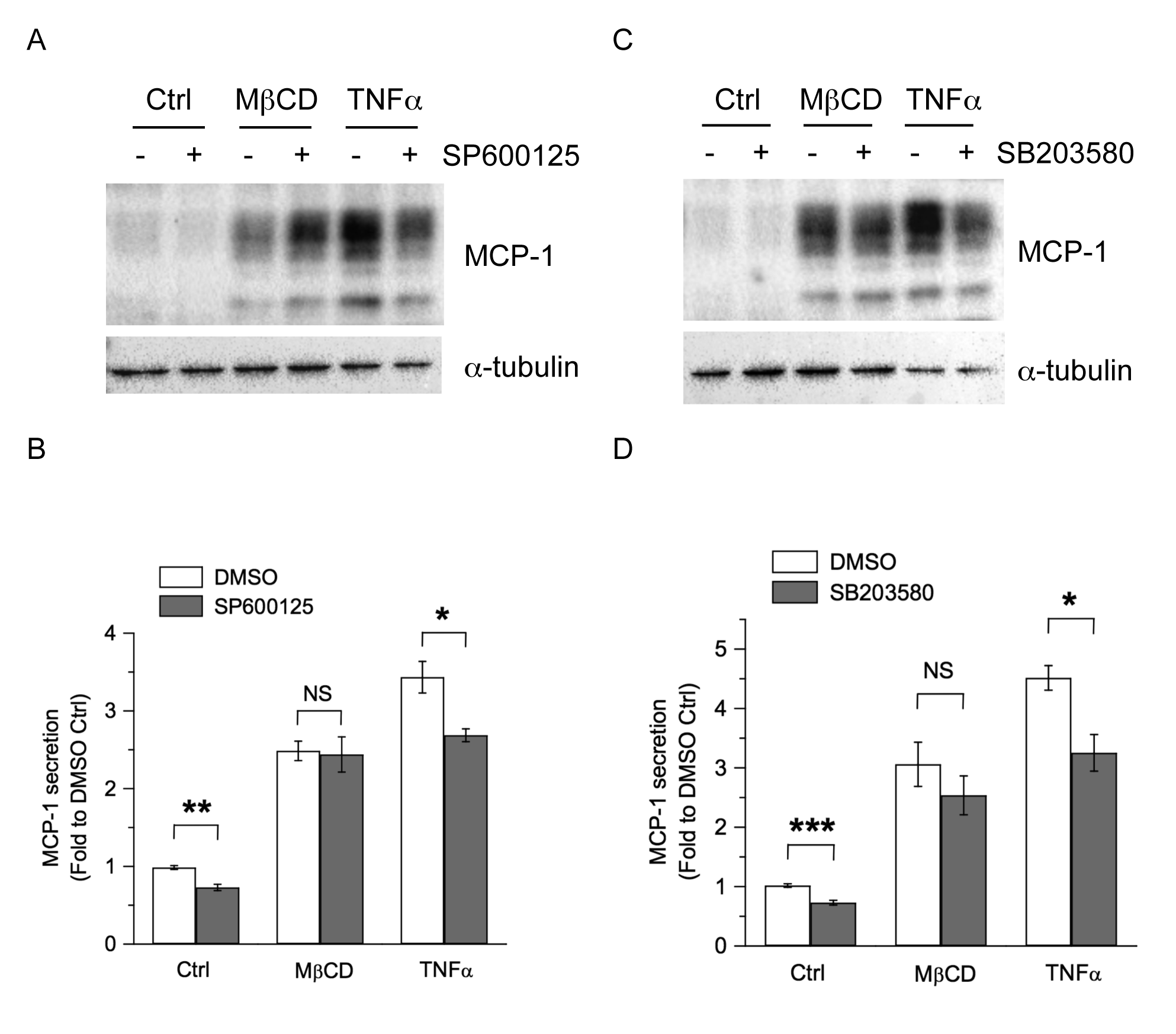

Supplement: S5 Fig — (A, B) 3T3-L1 adipocytes were untreated (Ctrl), or treated with 4 mM MβCD or 1 ng/ml TNFα, together without (DMSO) or with 10 μM SP600125 for 4 h. (A) Cellular proteins were solubilized and subjected to SDS-PAGE and Western blot analysis with the indicated antibodies. Representative immunoblots from three independent experiments are shown. (B) MCP-1 secretion in medium was determined by ELISA. Each point represents the mean ± S.E. of four independent experiments. Asterisks denote significant difference (*p<0.05, **p<0.01). NS, not significant. (C, D) 3T3-L1 adipocytes were untreated (Ctrl), or treated with 4 mM MβCD or 1 ng/ml TNFα, together without (DMSO) or with 10 μM SB203580 for 4 h. (C) Cellular proteins were solubilized and subjected to SDS-PAGE and Western blot analysis with the indicated antibodies. Representative immunoblots from three independent experiments are shown. (D) MCP-1 secretion in medium was determined by ELISA. Each point represents the mean ± S.E. of five independent experiments. Asterisks denote significant difference (*p<0.05, ***p<0.001). NS, not significant. (TIF) [file pone.0169005.s005.tif]

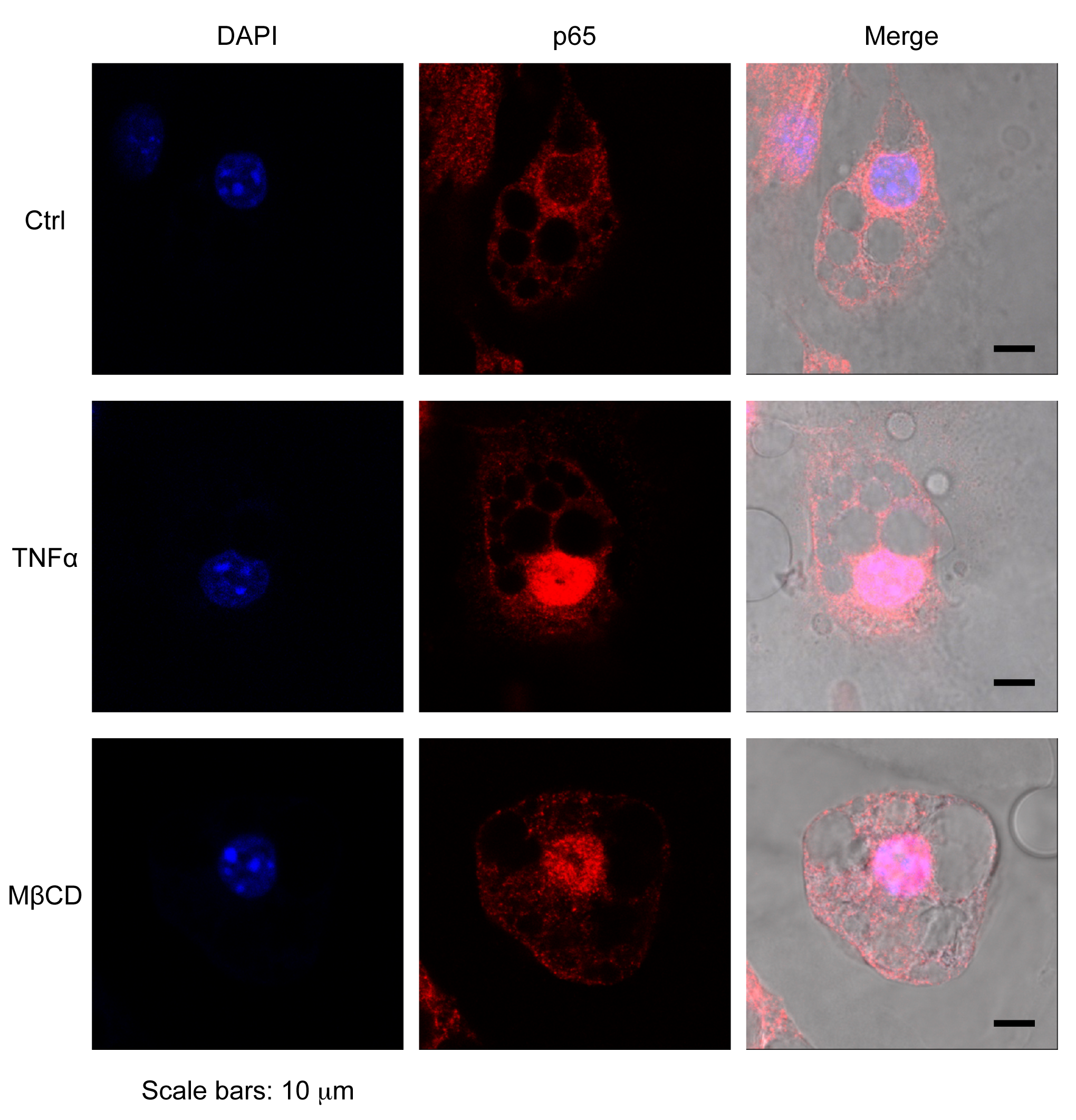

Supplement: S6 Fig — 3T3-L1 adipocytes were untreated (Ctrl), or treated with 4 mM MβCD or 1 ng/ml TNFα for 30 min. NF-κB localization was stained with anti-p65 NF-κB antibody (red). Nuclei were stained with DAPI (blue). Scale bar = 10 μm. (TIF) [file pone.0169005.s006.tif]

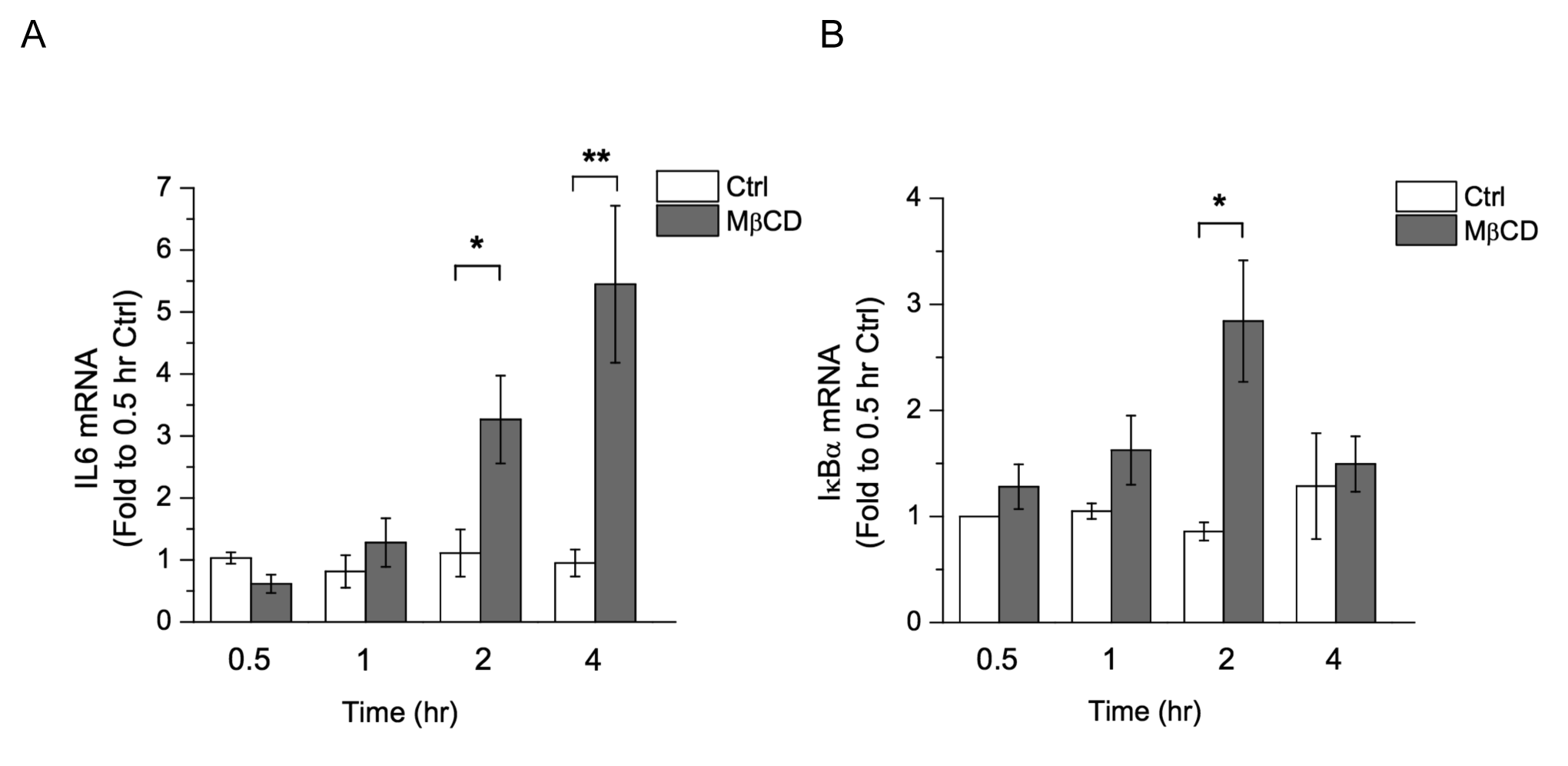

Supplement: S7 Fig — 3T3-L1 adipocytes were untreated (Ctrl) or treated with 4 mM MβCD for 0.5, 1, 2, or 4 h. mRNA levels of IL-6 (A) and IκBα (B) were determined by qPCR. Each point represents the mean ± S.E. of at least three independent experiments. Asterisks denote significant differences (*p<0.05, **p<0.01). (TIF) [file pone.0169005.s007.tif]

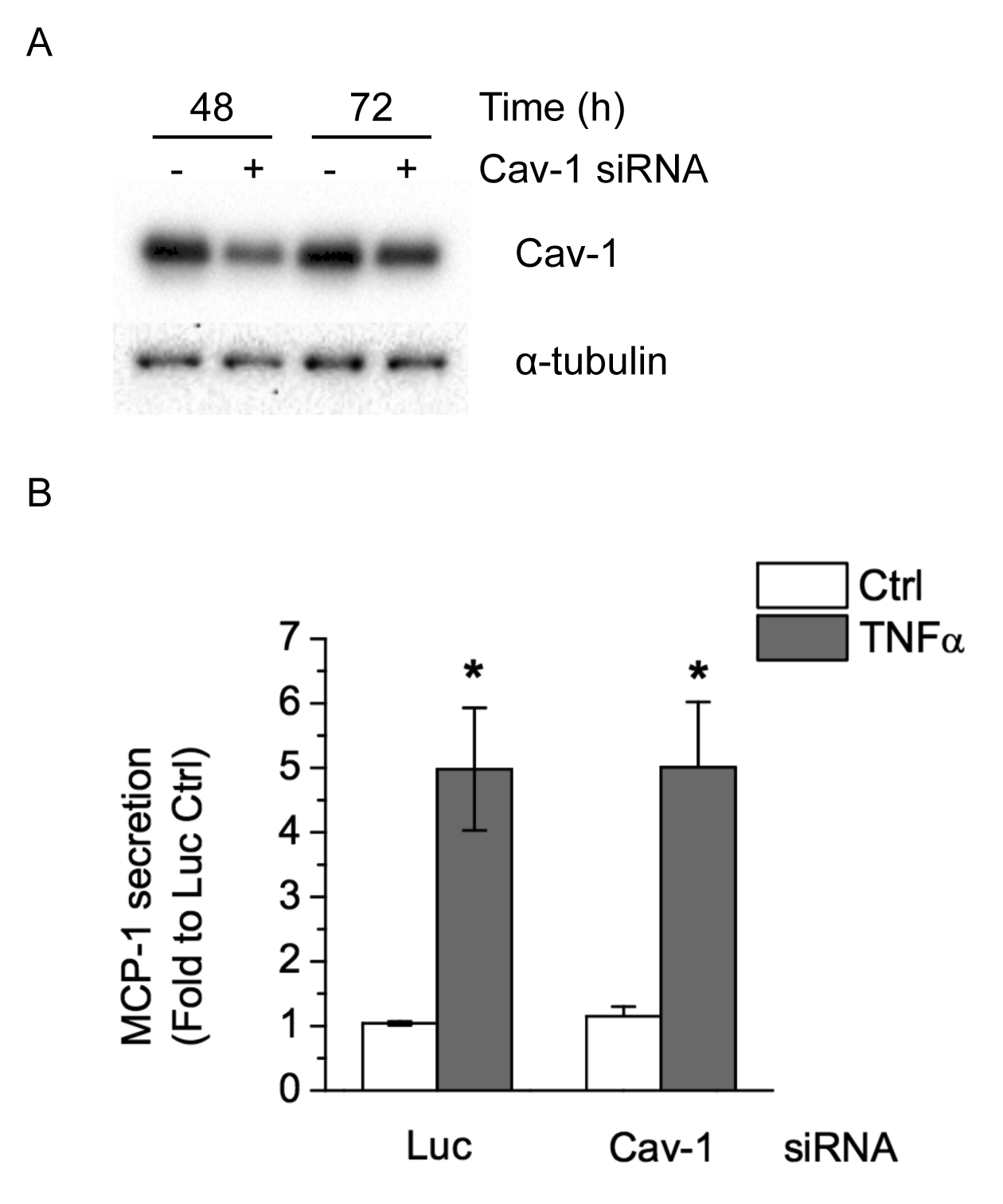

Supplement: S8 Fig — (A) 3T3-L1 adipocytes were transfected with non-targeting luciferase siRNA (-) or siRNA against caveolin-1 (+). Protein levels of caveolin-1 (Cav-1) were determined by Western blotting. (B) 3T3-L1 adipocytes were transfected with luciferase (Luc) or caveolin-1 (Cav-1) siRNA. 48 h post transfection, cells were untreated (Ctrl) or treated with 1 ng/ml TNFα for 4h. MCP-1 released into the media was determined by ELISA. Each point represents the mean ± S.E. of three independent experiments. Asterisks denote significant differences (*p<0.05, compared with Ctrl value). (TIF) [file pone.0169005.s008.tif]

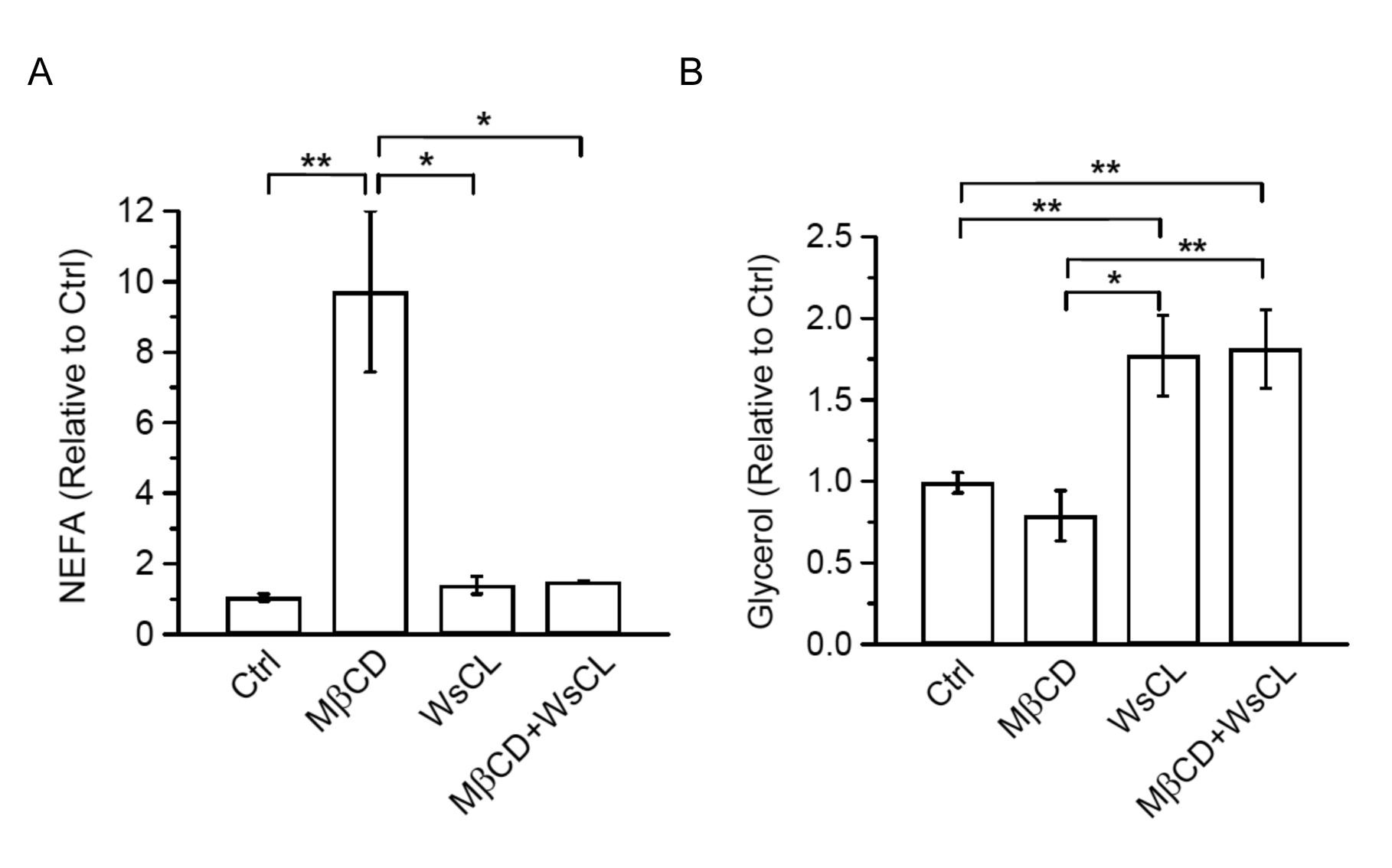

Supplement: S9 Fig — 3T3-L1 adipocytes were untreated (Ctrl), or treated with 4 mM MβCD, 250 μg/ml WSCL, or both for 24 h. Glycerol (A) or NEFA (B) release into the media and protein concentrations of cell lysate were determined. Each point represents the mean ± S.E. of at least three independent experiments. Asterisks denote significant differences (*p<0.05, **p<0.01). (TIF) [file pone.0169005.s009.tif]
