# Supplementary material for: Disruption of Lipid Raft Function Increases Expression and Secretion of Monocyte Chemoattractant Protein-1 in 3T3-L1 Adipocytes
Source: PLoS One. 2016 Dec 28;11(12):e0169005. doi: 10.1371/journal.pone.0169005 (PMC5193455; doi:10.1371/journal.pone.0169005)
Supplement: S1 File — (DOCX) [file pone.0169005.s010.docx]

**Supporting Information Methods**

**Indirect immunofluorescence** 3T3-L1 adipocytes were grown on coverslips with coating of poly-d-lysine (5 μg/cm^2^, BD Biosciences #354210). After treatment, cells were washed with phosphate buffer saline (PBS), fixed with 3.7% paraformaldehyde/PBS, and permeabilized with PBS containing 0.1% Triton X-100. Cells were incubated with the blocking solution (PBS containing 1% BSA) at room temperature for 1 h, and then incubated with anti-p65 antibody (1:2000) at 4^o^C. Cells were washed with cold PBS and incubated with Alexa Fluor 568-conjugated goat-anti-mouse antibody (1:2000) at room temperature for 1 h. The nucleus was stained with 0.1 μg/ml DAPI for 5 min at room temperature. Fluorescent images were taken with a Leica TCS SP5 Confocal Spectral Microscope Imaging System under a 100x oil-immersion objective.

**Cytotoxicity (MTT) assay** 3T3-L1 adipocytes were plated into 96-well plates. After treatment, cells were incubated with 100 μl MTT reagent [3- (4,5-cimethylthiazol-2-yl)-2,5-diphenyl tetrazolium bromide, 0.5 mg/ml in PBS] in the 37^o^C CO_2_ incubator for 4 h. After removal of the reagent, cells were lysed with 100 μl DMSO to dissolve the formazan, and the absorbance was read at 570 nm.

**Measurement of glycerol and NEFA** Glycerol and NEFA release in the medium was measured using a free glycerol reagent (Sigma, St Louis, MO) and LabAssay NEFA kit (#294-63601, Wako Pure Chemical Industries, Osaka, Japan), respectively, according to manufacturer’s specifications as described previously [[20](#_ENREF_20)]. In brief, 3T3-L1 adipocytes were incubated in 0.5 ml phenol red-free DMEM containing 3% BSA and the desired treatments. After centrifugation to remove cell debris, and the supernatants of cell media were divided into aliquots for the assays. For glycerol measurement, ten microliters of sample was incubated with the free glycerol assay reagent, and the absorbance was read at 540 nm. For NEFA measurement, four microliters of sample was incubated with reagents at 37^o^C, and the absorbance was read at 550 nm. A standard curve constructed from the glycerol or NEFA standards was used to calculate concentration in the culture supernatants. The cells remaining on the plate were washed and lysed in 1 N NaOH, and protein concentrations were measured and used to normalize the release value.
